# Sweat Wearable Sensor Based on Confined Pt Nanoparticles in 2D Conductive Metal–Organic Frameworks for Continuous Glucose Monitoring

**DOI:** 10.1002/advs.202507212

**Published:** 2025-06-23

**Authors:** Wei Huang, Yong Yang, Yun Xu, Fei Xiao, Lin Wang

**Affiliations:** ^1^ Key Laboratory of Material Chemistry for Energy Conversion and Storage Ministry of Education School of Chemistry and Chemical Engineering Huazhong University of Science & Technology Wuhan 430074 China; ^2^ School of Mechanical Science and Engineering Huazhong University of Science & Technology Wuhan 430074 China; ^3^ Union Hospital Tongji Medical College Huazhong University of Science and Technology Wuhan 430022 China

**Keywords:** confined metal nanoparticles, integrated wearable devices, microfluidic electrochemical biosensor, nonenzymatic glucose detection, phthalocyanine‐based metal‐organic framework

## Abstract

The noninvasive glucose sensors with comprehensive functional capabilities can enable wearable glucose monitoring in sweat with high sensitivity and minimal risk. However, the limited stability of natural enzymes, along with interference from electro‐oxidizable species, continues to pose significant challenges for their long‐term application. Herein, an integrated wearable system is presented for nonenzymatic glucose monitoring in sweat at the point of care. This system integrates a flexible microfluidic glucose sensor patch for sweat sampling and measurement, using Pt nanoparticles (Pt‐NPs) confined within phthalocyanine‐based conductive metal–organic frameworks (Pc‐MOFs) as electrode materials, and a flexible printed circuit board for signal/analysis and wireless communication. The microfluidic sensor patch based on Pc‐MOFs confined Pt‐NPs exhibits significantly improved nonenzymatic glucose sensing performances. This is attributed to the ultrasmall size of Pt‐NPs and the confinement effect within the Pc‐MOF channels, which regulates the glucose adsorption intensity and increases the electrocatalytic activity to glucose oxidation. During the continuous monitoring process, the glucose concentration is calibrated in sweat by accounting for fluctuations in pH and temperature, and evaluated the performance of the wearable device in monitoring sweat glucose levels in human subjects over a 12‐h period, achieving data as accurate as that obtained using high‐performance liquid chromatography.

## Graphical abstract

An integrated wearable system is developed for nonenzymatic sweat glucose monitoring at the point of care. The system couples a microfluidic patch based on Pt nanoparticles confined in phthalocyanine‐based conductive MOFs with a flexible wireless circuit. The confined ultrasmall Pt‐NPs within MOF channels enable high electrocatalytic activity and selective glucose adsorption.

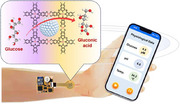

## 1 Introduction

Diabetes mellitus, a chronic metabolic disorder marked by persistently elevated blood glucose levels, has emerged as a global health crisis.^[^ ^1^, ^2^, ^3^ ^]^ If left uncontrolled, diabetes can result in severe complications such as cardiovascular disease, kidney failure, nerve damage, and vision loss.^[^ ^4^, ^5^ ^]^ Hence, the effective management of blood glucose levels is essential for preventing or delaying these potentially life‐threatening complications. The rapid development of wearable health monitoring devices has significantly advanced personalized medicine by providing real‐time, in vivo and non‐invasive insights into physiological parameters^[^ ^6^, ^7^, ^8^ ^]^ as several body fluids have emerged as promising mediums for non‐invasive blood glucose monitoring.^[^ ^9^, ^10^, ^11^ ^]^ Especially, the glucose concentration in sweat can closely correlate with blood glucose levels, rendering it an ideal candidate for glucose sensing.^[^ ^12^, ^13^ ^]^ Additionally, sweat glucose levels are influenced by various factors such as physical activity, diet, and stress, making sweat‐based glucose monitoring a valuable tool for real‐time health tracking.^[^ ^14^, ^15^ ^]^ The current wearable electrochemical sensors based on glucose oxidase (GOx) can offer high selectivity and sensitivity for glucose measurement. However, the limited stability of enzyme‐based sensors and the interference from electro‐oxidizable substances remain significant challenges.

To develop more effective glucose sensors for clinical and practical applications, it is essential to explore new electrode materials that enable direct glucose oxidation for non‐enzymatic sensing, as they can provides significant advantages in terms of high stability, enhanced sensitivity, improved selectivity, resistance to environmental fluctuations, and a longer operational lifespan that superior to those of the enzyme‐based sensors. Numerous non‐enzymatic glucose sensors have been investigated, with the majority developed in alkaline electrolytes.^[^ ^16^, ^17^, ^18^ ^]^ And the direct electrocatalytic oxidation of glucose under neutral conditions typically requires the use of precious metal nanoparticle (NP) catalysts, such as Au and Pt.^[^ ^19^, ^20^, ^21^, ^22^ ^]^ However, traditional precious metal based sensors frequently suffer from insufficient selectivity and sensitivity.^[^ ^23^, ^24^ ^]^ Therefore, to modify the nanostructure and composition of electrode materials is critical for improving the electrocatalytic performances of non‐enzymatic glucose sensors. The electrocatalytic glucose reactions usually take place at the catalyst's surface, where the availability of active sites and appropriate adsorption strength at the reaction interface are vital for optimal catalysis.^[^ ^25^ ^]^

In this work, we develop a phthalocyanine‐based conductive metal‐organic framework (Pc‐MOF) with pore sizes well‐matched to glucose molecules, enabling selective adsorption and enrichment at the electrode interface. The pores of Pc‐MOF also serve as a scaffold to confine ultrafine Pt‐NPs, thereby enhancing catalytic efficiency and regulating glucose–catalyst interactions. 2D conductive metal‐organic frameworks (c‐MOFs) have garnered significant attention due to their layered structures, characterized by strong in‐plane π‐conjugation and weak out‐of‐plane π–π interactions.^[^ ^26^ ^]^ These materials exhibit regular porosity, large specific surface area, excellent conductivity, diverse topological structures, and unique physicochemical properties. However, most reported redox‐active 2D c‐MOFs are constructed from redox‐inactive aromatic building blocks, such as benzene and triphenylene, which limit their catalytic centers to a single redox site.^[^ ^27^ ^]^ In contrast, Pc‐MOFs surpass traditional 2D c‐MOFs due to their exceptional properties, including superior electrical conductivity, multiple active redox sites derived from both metal ion centers and phthalocyanine ligands, and remarkable chemical and thermal stability, which collectively enhance their catalytic activity and long‐term durability, and broaden their potential for electrochemical sensing and catalysis.^[^ ^28^, ^29^, ^30^ ^]^ Confining Pt‐NPs within the well‐defined channels of Pc‐MOFs can significantly reduce the size of Pt‐NPs and increase the number of available active sites,^[^ ^31^ ^]^ and thereby improve the electrocatalytic performances of the resultant nanohybrid electrocatalyst toward the glucose oxidation reaction. Moreover, the confinement effect within the customized Pc‐MOF channels can promote the selective interactions between Pt‐NPs and glucose molecules as well as modulate the adsorption strength of glucose, giving rise to the improved selectivity, sensitivity, and stability for glucose detection in electrochemical sensing.

For clinical practical application, we further developed a fully integrated non‐invasive sweat monitoring system for continuous, real‐time tracking of blood glucose with minimal discomfort or risk. By integrating a flexible microfluidic electrochemical sensor patch for sweat sampling and measurement with a reusable, wireless, flexible printed circuit board for signal processing and transmission, this system offers a user‐friendly and convenient platform for sensitive detection of sweat glucose levels, with a linear response to glucose levels up to 800 µm in artificial sweat, a detection limit of 5.5 µm (S/N = 3) and a sensitivity of 25.48 µA cm^−2^ mM^−1^. During the continuous wearable monitoring process, we have calibrated the sweat glucose concentration by accounting for fluctuations in pH and temperature in human subjects over a 12‐h period of daily activities. In virtue of the superior sensing performances and capacity to account for physiological fluctuations, the proposed sensor device holds the potential to revolutionize non‐invasive glucose monitoring in both clinical and everyday settings, offering valuable insights for healthy individuals and patients with diabetes.

## 2 Results and Discussion

### 2.1 Synthesis and Characterization of Pt‐NPs/MnPc‐Mn

**Figure 1:**
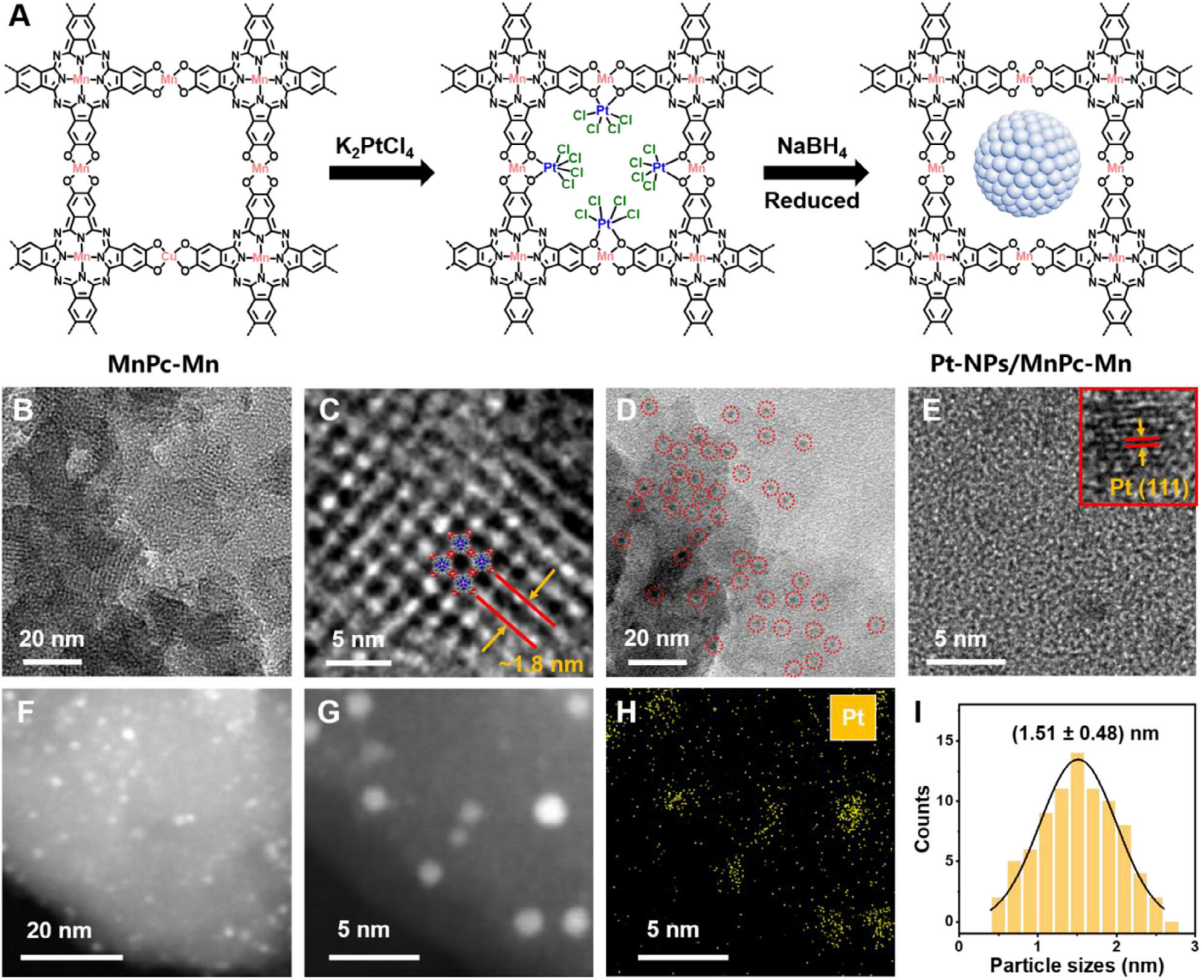
Synthesis and structure of Pt‐NPs/MnPc‐Mn. A) Schematic illustration for the preparation of Pt‐NPs/MnPc‐Mn. B) TEM image and C) HR‐TEM image of MnPc‐Mn. D) TEM image and E) HR‐TEM image of Pt‐NPs/MnPc‐Mn. Inset of E): the interplanar spacing of Pt (111) plane. F,G) HAADF‐STEM images of Pt‐NPs/MnPc‐Mn with different magnification. H) EDS elemental mapping image of Pt‐NPs in MnPc‐Mn. I) Particle size distribution of Pt‐NPs in MnPc‐Mn.

**Figure** **1**A outlines the synthesis process of Pt‐NPs/MnPc‐Mn. Initially, a dual‐active‐site Pc‐MOF (i.e., MnPc‐Mn) was synthesized using a hydrothermal approach with manganese (II) acetylacetonate and MnPc‐OH as precursors. The as‐obtained MnPc‐Mn has rigid channels with an average diameter of 2 nm, which provides numerous active sites and nano‐confined spaces for the nucleation and growth of Pt‐NPs. To prepare Pt‐NPs/MnPc‐Mn composite, K_2_PtCl_4_ was added to a suspension of MnPc‐Mn in deionized water. The metal ions interact with the oxygen groups in the organic linkers of MnPc‐Mn, forming dipolar interactions that stabilize the metal ions within the MOF pores. NaBH_4_ was then introduced to reduce the metal precursor, resulting in the formation of ultrafine Pt‐NPs (<2 nm) embedded inside the MnPc‐Mn structure.

Figure 1B shows the transmission electron microscopy (TEM) image of the synthesized MnPc‐Mn, displaying its characteristic layered morphology and 2D stacked structure, which closely resembles 2D nanosheets (e.g., graphene). The high‐resolution TEM image clearly shows square unit cells at the molecular level, with a lattice parameter of a = b = 1.8 nm (Figure 1C). Furthermore, as demonstrated in Figure 1D, the ultrafine Pt‐NPs, confined within the pores of the Pc‐MOF, are uniformly distributed throughout the MnPc‐Mn matrix (Figure S1, Supporting Information). The high‐resolution TEM image reveals a lattice spacing of 2.15 Å, corresponding to the Pt (111) crystal plane (Figure 1E). The high‐angle annular dark‐field scanning transmission electron microscopy (HAADF‐STEM) image further confirms the uniform distribution of Pt‐NPs in MnPc‐Mn (Figure 1F,G). The energy‐dispersive X‐ray spectroscopy (EDS) elemental mapping image reveals that Pt is evenly dispersed throughout MnPc‐Mn (Figure 1H). The average size of Pt‐NPs is 1.51 ± 0.48 nm (Figure 1I), which is slightly smaller than the ≈2 nm pore size of MnPc‐Mn. To optimize the Pt loading content, we synthesized MnPc‐Mn composites containing 5, 10, and 20 wt.% Pt under the same conditions. TEM images (Figure S2, Supporting Information) show that at 5 and 10 wt.% loadings, the diameters of the Pt clusters remain at ≈1.0 and ≈1.5 nm, respectively—well within the ≈2 nm pores of MnPc‐Mn—while the ≈5 nm particles produced by the 20 wt.% loading partially block the pores.

**Figure 2:**
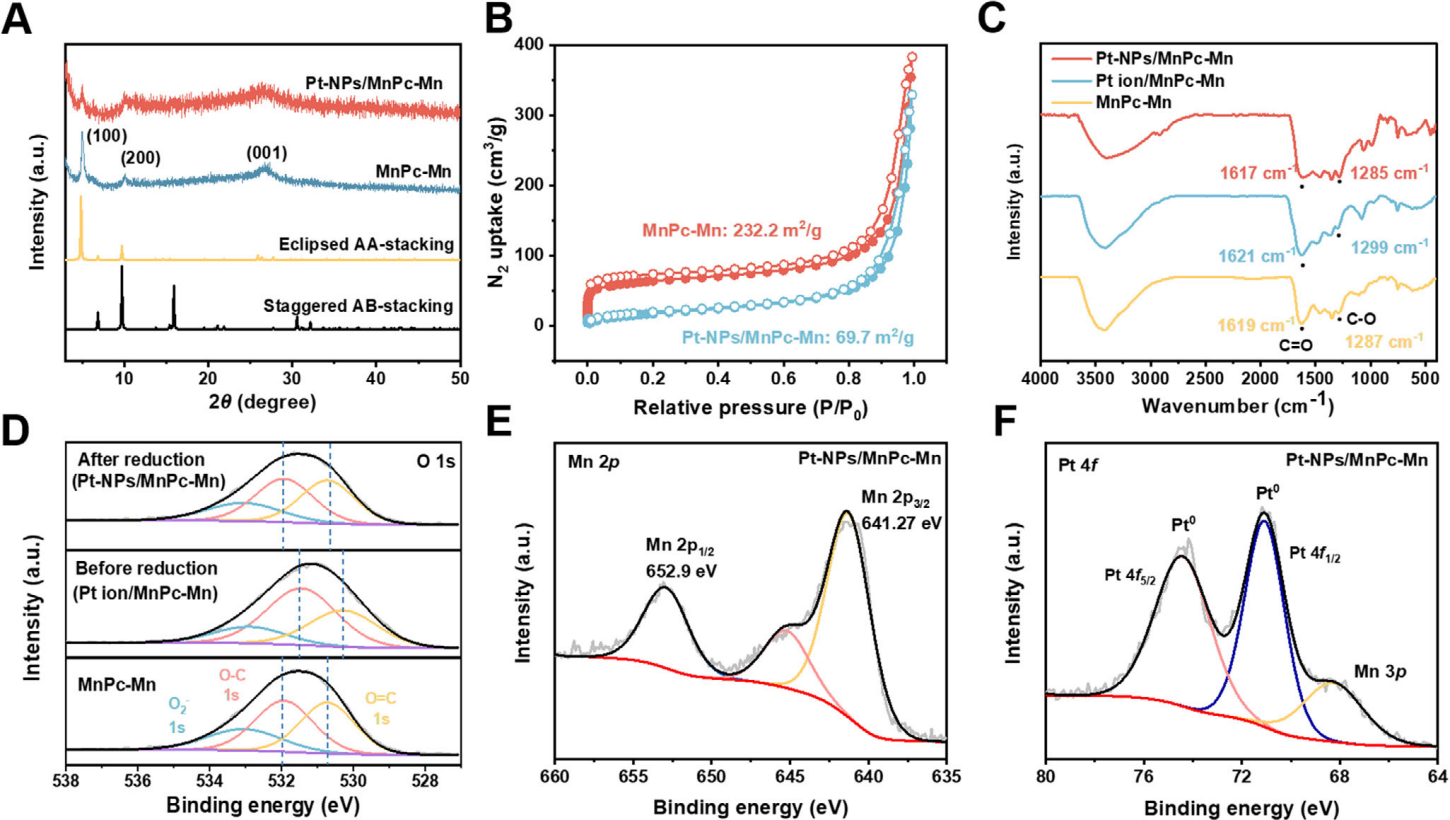
Characterization of Pt‐NPs/MnPc‐Mn. A) PXRD patterns of MnPc‐Mn and Pt‐NPs/MnPc‐Mn. B) N_2_ adsorption and desorption isotherms at 77 K. C) FT‐IR spectra of MnPc‐Mn and Pt‐NPs/MnPc‐Mn. D) Ex situ XPS spectra of O 1s regions in MnPc‐Mn, Pt ion/MnPc‐Mn and Pt‐NPs/MnPc‐Mn. E,F) High‐resolution XPS spectra of Mn 2p and Pt 4f regions in Pt‐NPs/MnPc‐Mn.

The crystalline of Pt‐NPs/MnPc‐Mn has been evaluated using powder X‐ray diffraction (PXRD) to investigate the structural integrity after the encapsulation of Pt‐NPs (**Figure** **2**A). The PXRD pattern of pristine MnPc‐Mn shows characteristic peaks for the (100), (200), and (001) planes, which are consistent with the structure of MnPc‐Mn.^[^ ^32^ ^]^ And these main peaks remain intact in the PXRD pattern of Pt‐NPs/MnPc‐Mn, suggesting that the Pc‐MOF framework is well‐preserved after Pt‐NPs encapsulation. Although the overall intensity of the MnPc‐Mn diffraction peaks diminishes upon Pt loading, the positions of the (100), (200), and (001) reflections remain essentially unchanged. This indicates that the long‐range order of the 2D framework is preserved even after NPs insertion. The slight peak broadening observed is primarily due to the introduction of ultra‐small Pt NPs within the pores, which increases microstrain and reduces coherent scattering domain size, rather than wholesale collapse of the MOF lattice. Furthermore, the N_2_ adsorption‐desorption experiments at 77 K confirm the inclusion of Pt‐NPs within the MnPc‐Mn pores (Figure 2B). Although the specific surface area of Pt‐NPs/MnPc‐Mn (69.7 m^2^ g⁻¹) is lower than that of pristine MnPc‐Mn (232.2 m^2^ g⁻¹) due to the blockage of pores and the contribution of non‐porous Pt‐NPs, Pt‐NPs/MnPc‐Mn still retains its microporous structure (type I isotherm) with a substantial surface area. After Pt‐NPs encapsulation, the pore volume of Pt‐NPs/MnPc‐Mn is also lower than 2 nm for MnPc‐Mn (Figure S3, Supporting Information), confirming that Pt‐NPs are stabilized within these 2 nm pores. The overall reduction in pore volume is attributed to the diminished interparticle spaces and the aggregation of Pt‐NPs in the MnPc‐Mn matrix. This suggests that the Pt‐NPs in the surface pores of the 2D Pc‐MOF effectively block some of the intrinsic pores and reduce the interparticle spaces. Fourier transform infrared (FTIR) spectroscopy shows a shift in the C─O stretching frequency from 1287 cm^−1^ in the pristine MnPc‐Mn to 1299 cm^−1^ after introducing Pt precursor ions (Figure 2C), indicating electron density transfer between the C─O groups and the metal precursor.^[^ ^33^ ^]^

To investigate the site‐specific nucleation of Pt‐NPs within MnPc‐Mn, ex situ X‐ray photoelectron spectroscopy (XPS) was conducted at different stages of Pt‐NPs formation (Figure 2D). After introducing the metal precursors to the Pc‐MOF (Pt ion/MnPc‐Mn), the O 1s binding energy of the organic linker in MnPc‐Mn, corresponding to O─C (531.94 eV) and O═C (530.72 eV) groups, shifts to lower binding energies of 531.46 eV for O─C and 530.27 eV for O═C.^[^ ^34^ ^]^ This shift is attributed to the electron density transfer from the metal ions to the oxygen atoms, indicating that the Pt precursor ions (PtCl₄^2^⁻) bind to the oxygen functionalities in the Pc‐MOF linker (*i.e*., MnPc‐OH).^[^ ^35^ ^]^ XPS analysis also confirmed the chemical compositions and states of MnPc‐Mn and Pt‐NPs/MnPc‐Mn. Both of them show four prominent peaks in the survey spectra at 285, 399, 532, and 642 eV, corresponding to C 1s, N 1s, O 1s, and Mn 2p, respectively (Figures S4 and S5, Supporting Information). The high‐resolution Mn 2p spectrum of Pt‐NPs/MnPc‐Mn displays two peaks at binding energies of 641.27 and 652.9 eV, attributed to Mn 2p_3/2_ and Mn 2p_1/2_, respectively, confirming that Mn exists solely in the +2 oxidation state (Figure 2E).^[^ ^36^ ^]^ The Pt 4f XPS spectra of Pt‐NPs/MnPc‐Mn reveal characteristic peaks at 71.1 and 74.5 eV, corresponding to Pt⁰ 4f_1/2_ and Pt⁰ 4f_5/2_, respectively (Figure 2F).

### 2.2 Electrochemical Sensing Performance of Pt‐NPs/MnPc‐Mn Toward Glucose

**Figure 3:**
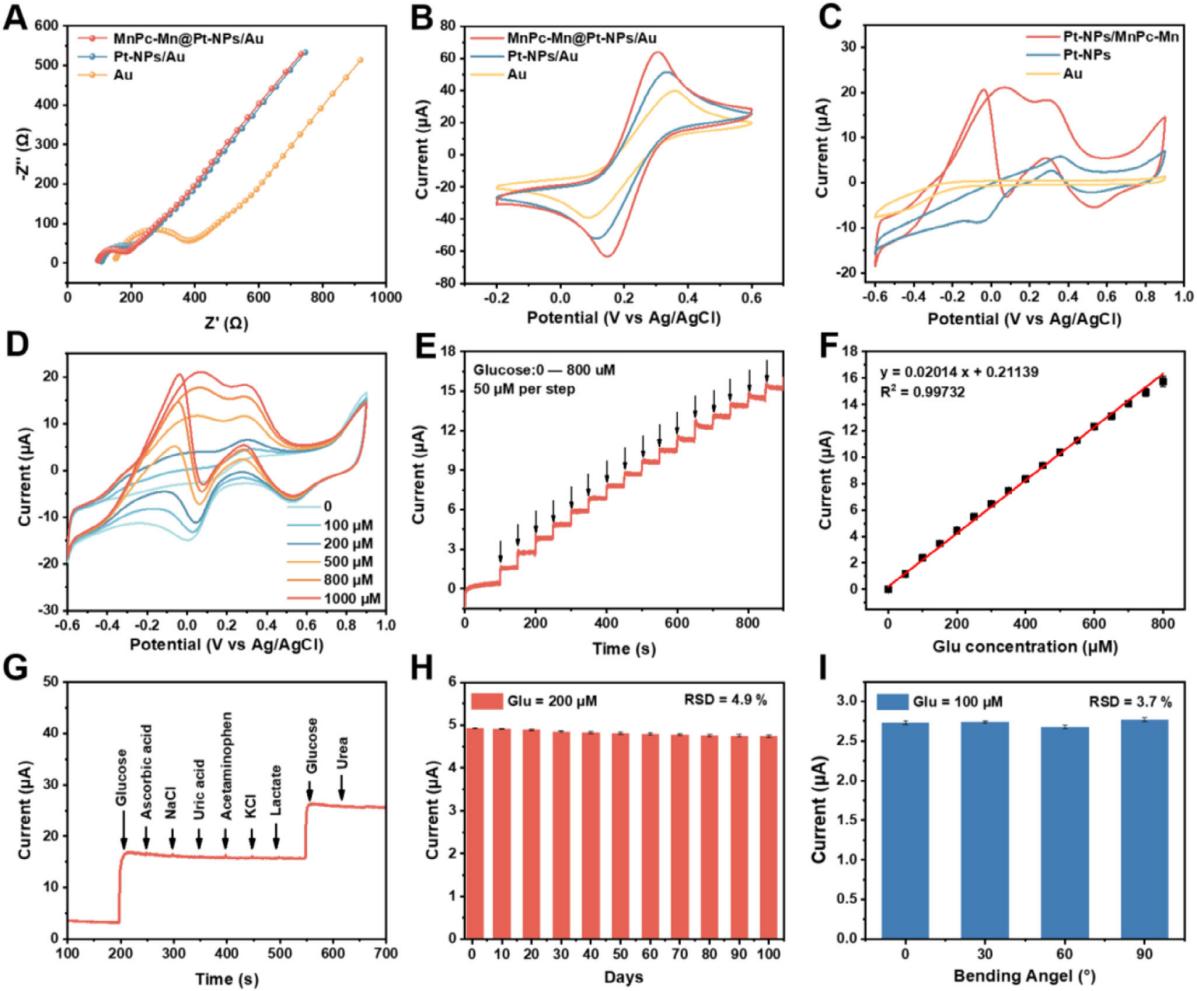
Electrochemical investigations of the proposed sensing system based on different electrodes. A) Nyquist plots and B) CV curves of Pt‐NPs/MnPc‐Mn/Au, Pt‐NPs/Au, and Au electrodes in 0.1 m KCl solution containing 5.0 mm [Fe(CN)_6_]^3−/4−^. C) CV curves of Pt‐NPs/MnPc‐Mn/Au, Pt‐NPs/Au and Au electrodes in 0.1 K PBS with 10 mm glucose. D) CV curves of the Pt‐NPs/MnPc‐Mn/NBG in 0.1 m PBS with increasing concentrations of glucose. Scan rate: 50 mV s^−1^. E) Amperometric responses of Pt‐NPs/MnPc‐Mn/Au electrode upon injecting the same aliquots of glucose in 0.1 m PBS. F) Linear calibration graph between the amperometric current and glucose concentration. G) Amperometric current response of Pt‐NPs/MnPc‐Mn/Au electrode to the addition of 800 µm glucose and 10 µm ascorbic acid, 10 mm NaCl, 1 mm uric acid, 0.1 mm acetaminophen, 10 mm KCl, 5 mm lactate, 500 µm glucose, and 0.1 mm urea in stirred 0.1 m PBS. Amperometric current response of the fully integrated sensor H) stored over 100 days toward 200 µm glucose and (I) at different bending angles.

Electrochemical impedance spectroscopy (EIS) was performed to investigate the interfacial behavior of the electrodes modified with different catalysts. **Figure** **3**A presents the Nyquist plots of Pt‐NPs/MnPc‐Mn/Au, Pt‐NPs/Au, and Au electrodes in 0.1 m KCl containing 5 mm [Fe(CN)_6_]^3−/4−^. The charge transfer resistance (*R*_ct_) values are estimated by calculating the diameter of the semicircle, which are found to be 92, 100, and 230 Ω for Pt‐NPs/MnPc‐Mn/Au, Pt‐NPs/Au, and Au electrodes, respectively. This highlights the superior electron transport capability of Pt‐NPs/MnPc‐Mn/Au in comparison to the other electrodes. Figure 3B illustrates that the cyclic voltammetry (CV) curves of all electrodes exhibit distinct symmetrical redox peaks attributed to the reversible single‐electron redox reaction of [Fe(CN)_6_]^3−/4−^. In comparison, the CV curve of Pt‐NPs/MnPc‐Mn/Au exhibits the lowest peak‐to‐peak potential separation (Δ*E*_p_) and the highest peak current in comparison to other modified Au electrodes, suggesting that Pt‐NPs/MnPc‐Mn electrocatalyst has significantly accelerated the heterogeneous electron transfer between the electrode and the [Fe(CN)_6_]^3−/4−^ redox species, and increased the effective electroactive surface area as well. This is attributed to the dual redox architecture of MnPc‐Mn—combining Mn‐N_4_ phthalocyanine linkers and Mn‐O4 metal nodes—which creates adjacent catalytic sites, reduces the oxidation overpotential, and enables fast electron delocalization together with the π‐conjugated framework.

Figure 3C displays the CV profiles of Pt‐NPs/MnPc‐Mn/Au, Pt‐NPs/Au, and Au electrodes in oxygen‐free PBS (pH 7.4) containing 1.0 mm glucose. For Pt‐NPs/MnPc‐Mn/Au, multiple oxidation peaks associated with glucose oxidation and its intermediates are observed at ≈0 and ≈0.3 V during the positive scan, while a prominent oxidation peak appears at ≈ −0.1 V during the negative scan. These observations are consistent with the competitive adsorption model, in which glucose and common interferents (e.g., chloride ions, ascorbic acid, uric acid) vie dynamically for the same Pt‐based active sites, their relative adsorption governed by differences in affinity.^[^ ^37^, ^38^ ^]^ By comparison, the CV curve of Pt‐NPs/Au electrode shows significantly reduced hump‐like current peaks arising from electroadsorbed glucose enediol intermediates at ≈0 V, along with much lower peak current densities for surface reactive species. In addition, the CV curve of Au electrode does not exhibit any observable peaks. Upon glucose addition, the oxidation current of Pt‐NPs/MnPc‐Mn/Au increases linearly. These demonstrate extremely high electrocatalytic activity of Pt‐NPs/MnPc‐Mn toward glucose oxidation (Figure 3D). The superior electrocatalytic activity can be largely ascribed to the unique structural characteristics of the MnPc‐Mn framework. As a 2D phthalocyanine‐based MOF, MnPc‐Mn possesses strong in‐plane π–d conjugation, which enhances its electrical conductivity and supports rapid electron transfer at the electrode surface. Particularly, the uniform pore size of ≈2 nm closely matches the molecular size of glucose, enabling size‐selective adsorption. This structural compatibility allows for effective enrichment and pre‐concentration of glucose near the active centers, enhancing reaction efficiency. Meanwhile, the high surface area ensures abundant exposure of Mn‐N_4_ and Mn‐O_4_ redox sites. These well‐defined channels also act as nanoreactors to spatially confine Pt NPs, preventing aggregation, improving catalytic site availability, and promoting synergistic interactions between Pt and the MnPc‐Mn matrix. Chronoamperometry (Figure S6, Supporting Information) shows the 10 wt.% sample delivers the highest steady‐state glucose‐oxidation current, while the 20 wt.% electrode exhibits increased noise and no further sensitivity gain due to loss of confinement. This systematic Pt‐loading study confirms that precise pore–particle matching (≈1.5 nm Pt in ≈2 nm channels) is critical to maximize active‐site density, enhance substrate pre‐adsorption, and boost turnover in our Pt‐NPs/MnPc‐Mn sensor.

Figure 3E shows the amperometric responses of Pt‐NPs/MnPc‐Mn/Au electrode to successive 50 µm glucose injections at an applied potential of 0 V. By operating at 0 V, interference from common electroactive species—such as ascorbic acid and uric acid, which oxidize at potentials above +0.3 V—is effectively suppressed, thereby enhancing the sensor's selectivity under physiological conditions. The corresponding calibration curve in Figure 3F reveals a sensitivity of 25.48 µA mM^−1^ cm^−2^ over a linear range of 0≈800 µm, with an excellent correlation coefficient (R^2^ = 0.9972). This range can encompass the typical sweat glucose concentrations which vary from 6 to 300 µm. The calculated limit of detection (LOD) is 5.5 µm (S/N = 3), surpassing most nanocatalyst‐based, nonenzymatic glucose sensors operating under physiologically relevant, neutral conditions in recent literature. (Table S1, Supporting Information). The improved sensing performances of Pt‐NPs/MnPc‐Mn are attributed to abundant dual‐redox sites in MnPc‐Mn and the ultrafine Pt‐NPs confined within its pores, which significantly increase the number of active sites and enhance the atomic utilization of noble metals, and promote the electrocatalytic activity toward the glucose oxidation reaction. Furthermore, the confinement effect within the customized Pc‐MOF channels can promote the selective interactions between Pt‐NPs and glucose molecules as well as modulate the adsorption strength of glucose, which not only improves the sensitivity for nonenzymatic glucose detection, but also enhances the selectivity and stability of the resultant Pt‐NPs/MnPc‐Mn based electrochemical sensor.

To verify that our confinement strategy preserves both the MOF scaffold and the dispersion of Pt NPs under prolonged operation, we recovered Pt‐NPs/MnPc‐Mn from Au‐foil electrodes after 100 consecutive CV cycles in 0.1 m PBS (pH 7.0) containing 1 mm glucose. High‐resolution TEM (Figure S7, Supporting Information) reveals that the ultrasmall Pt NPs remain uniformly distributed within the ≈2 nm channels of the MnPc‐Mn framework, with no signs of agglomeration or migration. Corresponding PXRD patterns (Figure S8, Supporting Information) show that the characteristic MnPc‐Mn reflections at 2θ = 5.1°, 10.0°, and 26.7° retain both their positions and relative intensities, indicating that the long‐range order and crystallinity of the 2D MOF are preserved. Together, these ex situ analyses confirm that our Pc‐MOF's robust coordination environment effectively “locks” the Pt nanoparticles in place—even after 100 CV cycles—thereby maintaining over 90% of the initial glucose‐oxidation sensitivity and demonstrating exceptional structural stability.

The interference of various biomolecules and ions that are commonly in sweat (e.g., such as ascorbic acid, uric acid, acetaminophen, lactate, urea, NaCl, and KCl) on glucose detection has been investigated through amperometric measurements (Figure 3G). The results show that the addition of 800 µm glucose produced a rapid and significant current response, which was almost unaffected by the presence of other interferents, demonstrating the preferential selectivity of Pt‐NPs/MnPc‐Mn/Au for glucose detection. To evaluate its long‐term stability, the current response to 200 µm glucose was monitored at regular intervals. After 100 days, 93% of the initial current value was retained, indicating that the electrochemical sensor based on Pt‐NPs/MnPc‐Mn/NBG/Au exhibits excellent long‐term stability (Figure 3H). This enhanced durability arises from the robust metal–ligand coordination in the MnPc–Mn framework, which tightly chelates each Mn center and “locks” Pt NPs within the ≈2 nm channels, effectively preventing metal‐ion leaching and NP migration even under prolonged electrochemical cycling. This performance surpasses that of most recently reported glucose sensors (Table S2, Supporting Information). The exceptional long‐term stability of the Pt‐NPs/MnPc‐Mn sensor stems from the molecular‐scale confinement of Pt‐NPs within the uniform ≈2 nm pores of the MnPc‐Mn framework. This confinement effectively suppresses nanoparticle migration and aggregation, preserving the active surface area over extended cycling. Figure 3I presents the amperometric responses of the sensing patch at different bending angles, with a relative standard deviation (RSD) of 3.7%, demonstrating the high mechanical stability of the integrated sensor devices for wearable use on the skin across various body parts (Figure S9, Supporting Information).

### 2.3 Performances of pH and Temperature Integrated Sensor

**Figure 4:**
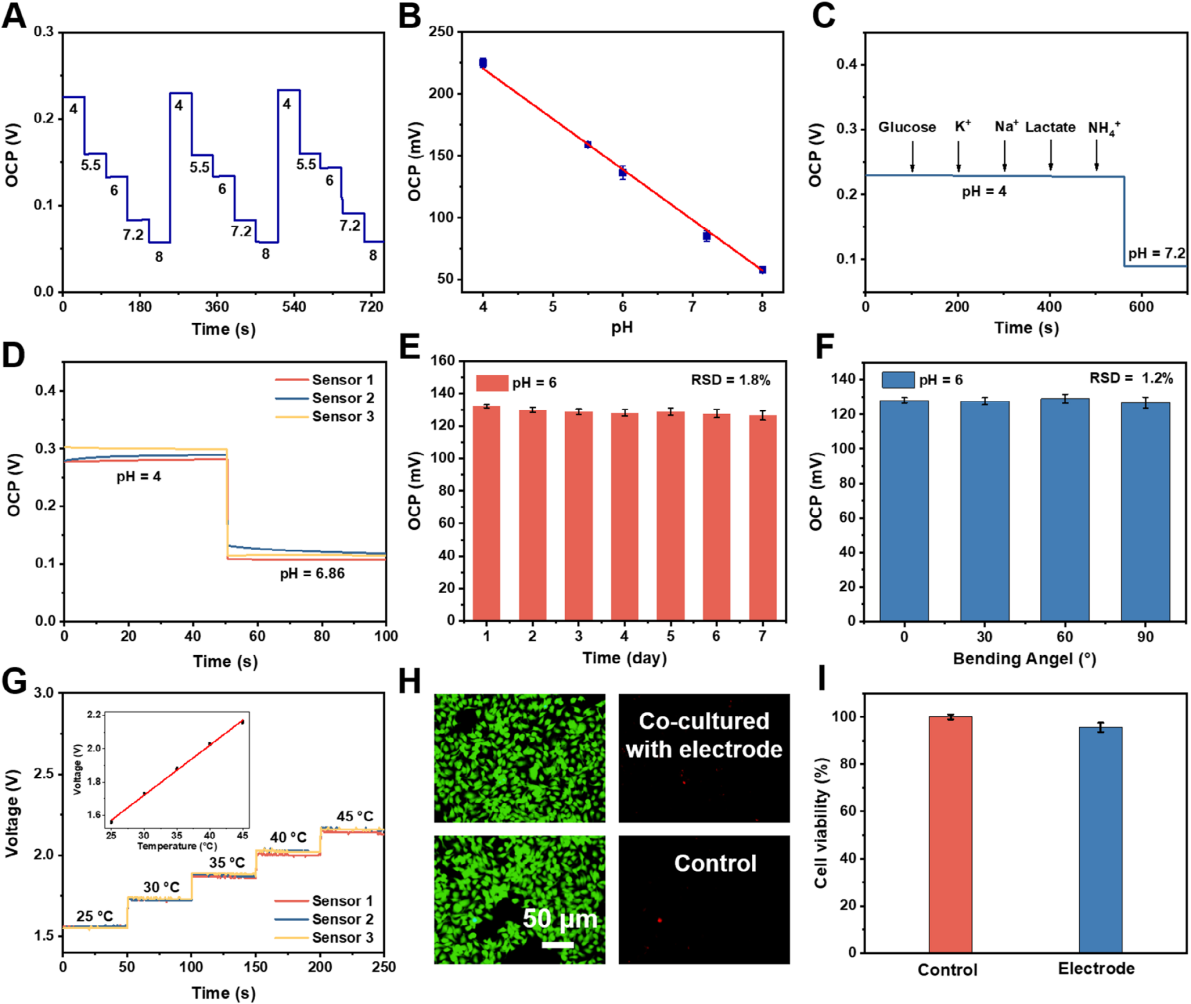
Electrophysiological characterization and biocompatibility test of the sensor patch. A) OCP vs. time profiles of the PANI‐based pH sensor. B) Calibration curve of the pH sensor. C) OCP response of the pH sensor after successive additions of 1.0 mm interference species in 0.1 m PBS. D) OCP vs. time curves for three pH sensors fabricated using the same process. E) The variation curve of OCP values over time. F) OCP response of the pH sensor at different bending angles. G) Voltage response of the temperature sensor. Insets: Linear relationship between voltage and temperature. H) Fluorescence microscopy images of live/dead assay of HACAT cells co‐cultured with sensor patch and without sensor patch (control) after 72 h immersion. I) Cell viability of the sensor patch and control.

The pH sensor was developed based on the protonation/deprotonation reactions of PANI under varying pH conditions, leading to changes in its conductivity. The performance of the PANI‐based pH integrated wearable sensor patch was evaluated using standard buffer solutions with pH values ranging from 4 to 8, demonstrating excellent open circuit potential (OCP) responses after repeated testing (3 iterations) at different pH levels (**Figure** **4**A). Figure 4B shows that the sensor exhibits a sensitivity of 40.793 mV pH^−1^, as calculated from the slope of the linearity plot, with a high linearity (R^2^ = 0.9951). The sensor's performance was enhanced by the phases of PANI and its deposition method, which further boosts the sensitivity. The pH sensor also exhibits excellent selectivity (Figure 4C), showing minimal response to other ions, such as glucose, K⁺, Na⁺, lactate, and NH₄⁺, while responding strongly only to H^+^. This high selectivity is attributed to the deprotonation of H^+^ on PANI surface.^[^ ^39^ ^]^ The reproducibility of the sensor was assessed by testing three randomly selected pH electrodes under identical conditions using buffer solutions at pH 4≈6.86, yielding an RSD value less than 5% for electrode‐to‐electrode consistency (Figure 4D). Figure 4E demonstrates the long‐term stability of the sensor, with an RSD value of 1.8% over 7 days of successive testing at pH 6, indicating minimal degradation in performance. Figure 4F presents the OCP responses at various bending angles, with an RSD value of 1.2%, highlighting the high flexibility, stability, and adaptability of the pH sensor for continuous monitoring during physical activity.

In order to conform to the skin's surface, we developed a flexible NiMn_2_O_4_‐based temperature sensor and incorporated it into the wearable patch. As the temperature increases, the resistance of the NiMn_2_O_4_ rises due to the enhanced atomic vibrations within the metal conductor, which impedes the electron flow. The dynamic resistance changes of these three sensors were recorded under gradual temperature increments from a constant temperature controller, as shown in Figure 4G. The sensor exhibits accurate and consistent performance across the physiological temperature range, with an impressive sensitivity of 0.003 °C^−1^ and a strong correlation coefficient (R^2^ = 0.99787), as illustrated in Figure 4G inset.

For practical sensing applications, we evaluated the biocompatibility of the integrated sensor patch through a cytotoxicity study using human immortalized keratinocyte cells (HACAT). A fluorescence double‐staining assay (calcein acetoxymethyl ester/propidium iodide, Calcein‐AM/PI) was conducted to assess the cell viability, which revealed strong green fluorescence from the live cells, with negligible red fluorescence from dead cells, after 72 h of incubation with the integrated sensor patch (Figure 4H). This indicates favorable biocompatibility of the sensor patch with the surrounding cells. Furthermore, the quantitative cytotoxicity tests using the CCK‐8 assay demonstrated over 90% cell viability after 72 h of co‐culture with the sensor patch (Figure 4I), confirming that the integrated sensor patch can be safely used in wearable sensing applications without biological safety concerns.

### 2.4 Design and Fabrication of Fully Integrated Wearable Sensor for Multi‐Modal Monitoring

**Figure 5:**
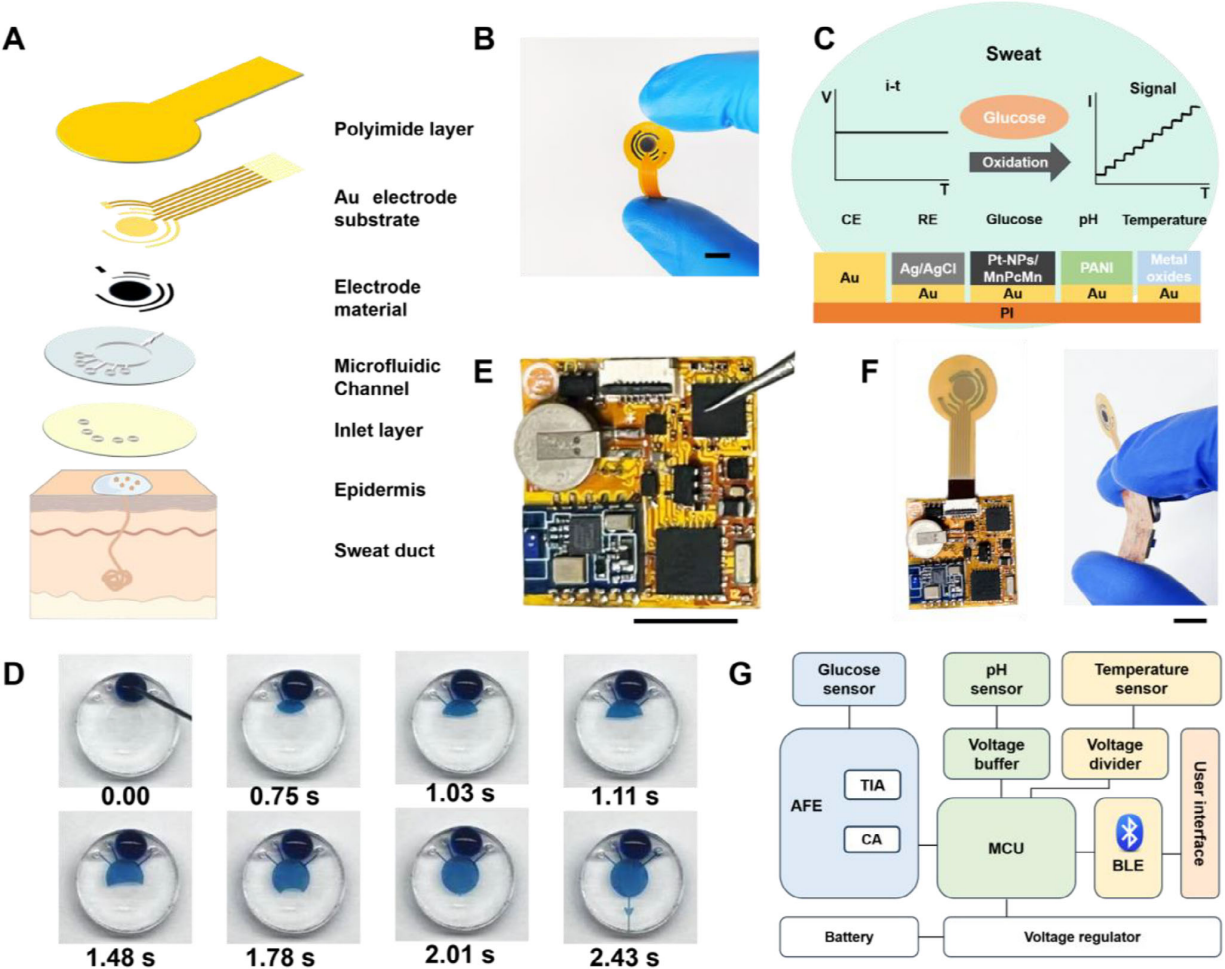
Schematics of the fully integrated wearable sensor for glucose management. A) Sensor layer structure including a modified electrode layer for sweat analysis, a microfluidic chip for sweat transport, and an inlet layer. B) Photograph of a disposable integrated sensor patch with Au electrode substrate. Scale bar: 1 cm. C) Scheme of the glucose, pH, and temperature sensors integrated sensing module. D) Photographs illustrating microfluidic sweat sampling. Photograph of E) the FPCB and F) the fully integrated flexible wearable sensor. Scale bar: 1 cm. G) System‐level block diagram of the FPCB.

**Figure** **5**A provides an overview of the fully integrated flexible wearable sensor designed for continuous monitoring of glucose, pH, and temperature in sweat. The key components of the integrated wearable device include chemical sensors, physical sensors, and a multi‐inlet microfluidic module. The system consists of five layers: a multiplexed electrode substrate plated on a polyimide base, different modified electrode materials for specific sensing functions, a patterned polydimethylsiloxane (PDMS) microfluidic channel, and a polyethylene terephthalate (PET) inlet layer. And the resultant flexible integrated sensor chip has been shown in Figure 5B. Figure 5C illustrates the design of the sensor patch integrated with different modified electrodes. The glucose sensor has utilized Pt‐NPs/MnPc‐Mn for direct electrooxidation of glucose in glucose biosensing. The pH sensor operates under varying pH conditions, leading to changes in its conductivity. The temperature sensor relies on the variation in electrical conductivity of NiMn_2_O_4_ with temperature. The microfluidic chip is designed to enable continuous sampling and excretion of human biofluids, ensuring constant renewal. The results show that the time required for sweat to reach 100% of the new solute concentration within the microfluidic chip is ≈2.43 s (Figure 5D). Additionally, it has been demonstrated that a sweat volume of just 2.5 µL is sufficient to completely fill the microchannel (Table S3, Supporting Information).

To enable wireless wearable sensing, a reusable flexible printed circuit board (FPCB) is fabricated using a polyimide (PI) substrate (Figure 5E). A disposable integrated patch can then be connected to the FPCB, forming a fully flexible and integrated wearable sensor (Figure 5F). The FPCB incorporates a microcontroller, voltage regulator, digital‐to‐analog converter (DAC), analog‐to‐digital converter (ADC), and Bluetooth module, facilitating efficient signal transduction, processing, and wireless transmission (Figure 5G). The weight of the fully integrated wearable sensor is only 0.8 g (Figure S10, Supporting Information). Relative to a standard over‐the‐counter glucose meter (≈$50), our integrated system costs only ≈50%, underscoring its exceptional affordability. (Tables S4 and S5, Supporting Information).

Since the performance of electrochemical sensors is influenced by changes in temperature and pH, we calibrated the integrated wearable sensor using calibration curves under varying pH and temperature conditions. The pH of sweat varies from person to person, typically ranging between 4.5 and 7, while the body surface temperature fluctuates between 27 and 38 °C. Figure S11 (Supporting Information) illustrates the amperometric response of the integrated wearable sensor in artificial sweat with different pH levels and its corresponding calibration curves. The results demonstrate that the direct oxidation current response of glucose on Pt‐NPs/MnPc‐Mn based nonenzymatic sensor is notably dependent on pH, as the slope of the calibration curve increases with rising pH. This behavior can be explained by the glucose oxidation mechanism, where the involvement of OH^−^ is essential for the oxidation process (Figure S12, Supporting Information).^[^ ^40^ ^]^ Similarly, Figure S13 (Supporting Information) shows the amperometric response of the sensor to glucose in artificial sweat at varying temperatures. The data reveal that the sensitivity of the sensor improves with increasing temperature, as higher temperatures can enhance the catalytic activity of the nanomaterials.^[^ ^41^ ^]^ However, higher temperatures also lead to increased signal noise. To account for the effects of pH and temperature, we applied correction factors to the amperometric response, enabling more accurate glucose quantification in human sweat (Figure S14, Supporting Information). The details of the glucose biosensor calibration for pH and temperature variation can be found in the Supporting Information.

### 2.5 Evaluation of the Wearable Sensor for Multiple Analysis in Human Subjects

**Figure 6:**
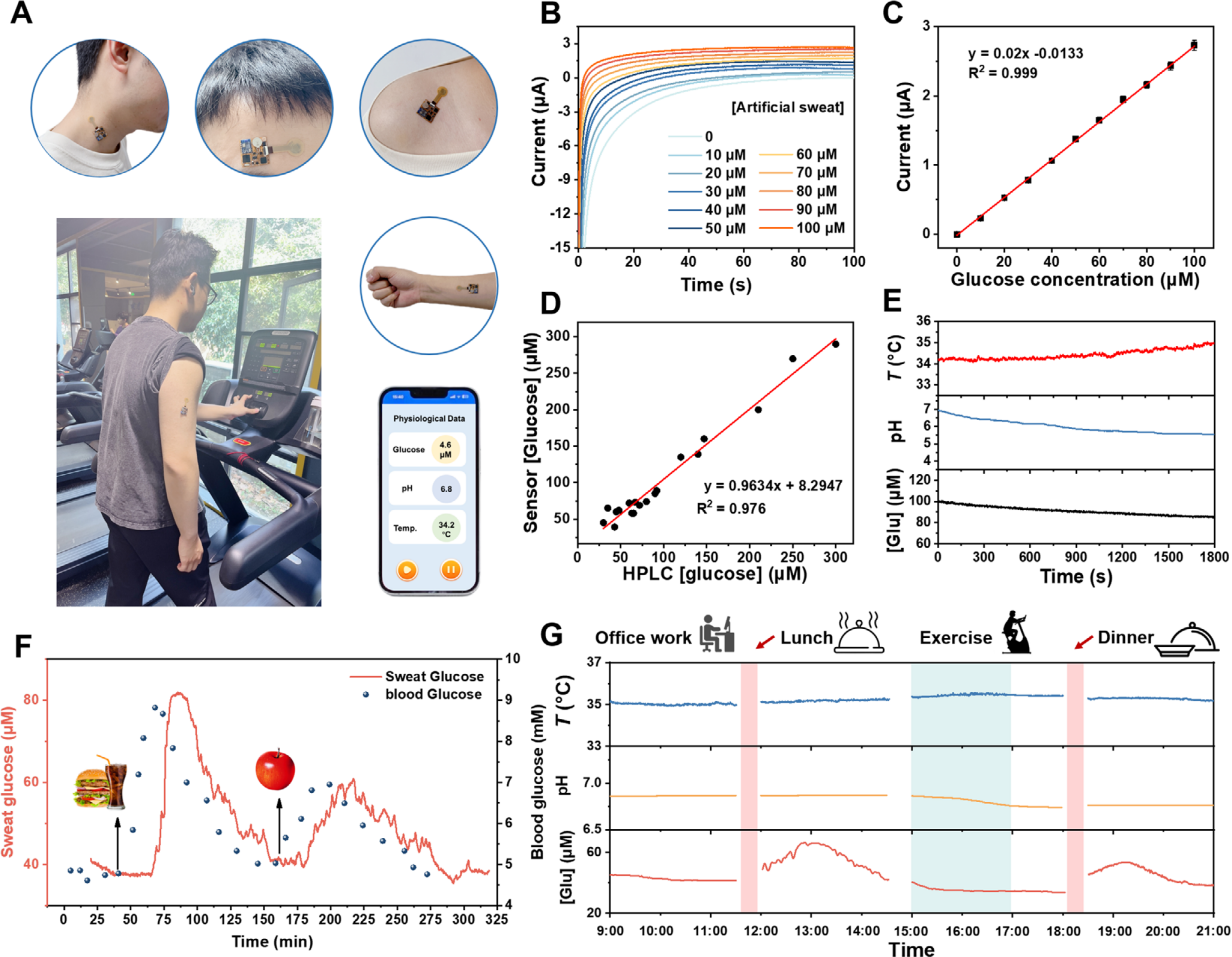
In vivo system validation of the sensor on the skin. A) Photographs of a healthy subject wearing the sensor patch at different body parts. B) Glucose detection with the fully integrated wearable sensor and C) the corresponding calibration plot. D) Correlation between glucose measured by HPLC and using sweat sensors for 19 sweat samples. E) Real‐time, continuous in situ measurement of temperature, pH, and sweat glucose levels from the arm of a healthy subject during a constant‐load jogging exercise. F) Multi‐event study of the glucose sensor involving varying food consumption events. G) Full‐day physiochemical surveillance of a subject while performing various activities.

The integrated wearable sensor can be comfortably worn on sweat‐producing areas of the skin across various parts of the body to fulfill the continuous monitoring of temperature, pH, and sweat glucose (**Figure** **6**A), demonstrating its feasibility for wireless, real‐time, in vivo long‐term monitoring. As shown in Figure 6B, the amperometric curves of the sensor have been recorded for artificial sweat containing varying low concentrations of glucose, with their corresponding calibration curve displayed in Figure 6C. The results show a linear increase in amperometric current with the addition of glucose, with a high correlation (0.999) over the entire calibration range from 0 to 100 µm. The wearable sensor demonstrates excellent regression coefficients, reflecting its high sensitivity and accuracy in detecting glucose levels in human sweat. Figure 6D shows the correlation between glucose measurements from high‐performance liquid chromatography (HPLC) and those obtained using the sweat sensor for 18 sweat samples, confirming the accuracy of the proposed glucose sensor.

The real‐time measurements of sweat glucose, pH, and temperature have been performed on the arm of a healthy subject during a jogging exercise (Figure 6E). During exercise, body temperature increased, while sweat pH decreased due to the secretion of lactic acid. Meanwhile, the glucose concentration in sweat declined due to glucose consumption in the blood. Figure 6F shows the integrated wearable sensor's response to multiple events, while commercial blood‐glucose test strips were used in parallel to continuously track dynamic changes in blood‐glucose levels (Figure S15, Supporting Information). We computed the Pearson correlation between sweat and blood glucose measurements across all five subjects over the 12‐h monitoring period, yielding r = 0.968 (95% CI: 0.946–0.984, p < 0.001). This strong, statistically significant correlation—accounting for repeated measures—confirms a robust linear relationship between the two data sets (Figure S16, Supporting Information). The results indicate that the glucose concentration in sweat is closely related to food consumption. Therefore, we have used the proposed sensor for continuous multimodal sensing to assess an individual's health in real‐time. Figure 6G presents 12‐h continuous multimodal monitoring during various daily activities. We recorded the stable sensor responses when the subject was in a fasting state and engaged in sedentary activities in the morning. After meals (e.g., lunch and dinner), the glucose levels rose ≈20 to 30 min post‐consumption, peaking ≈75 to 90 min, then gradually decreasing. During jogging, a decrease in pH and an increase in body temperature were also observed.

## 3 Conclusion

In conclusion, we proposed a wearable nonenzymatic glucose sensor based on highly active Pt‐NPs/MnPc‐Mn nano‐electrocatalyst for continuous, real‐time glucose monitoring in sweat. This fully integrated wearable sensor combines multimodal sensing capabilities for various physicochemical biomarkers. The electrode materials are custom‐designed and optimized to enable specific sensing functions. This approach, leveraging FPCB customization technology and the modification of functional nanomaterials, facilitates efficient and mask‐free patterning of a 2D architecture, meeting all manufacturing requirements for integrated wearable devices. We have also demonstrated the practical application of this sensor for real‐time health monitoring during daily activities. We envision that the proposed wearable electrochemical sensing device could enable continuous glucose monitoring and be used for the facile, low‐cost regular remote health tracking and clinical applications.

## 4 Experimental Section

### Synthesis of MnPc‐Mn

Manganese(II) 2,3,9,10,16,17,23,24‐octa‐hydroxyphthalocyanine (MnPc‐OH, synthetic details in Supporting Information) (35 mg, 0.05 mmol) was dispersed in 30 mL of N,N‐dimethylformamide (DMF), and 4 mL of 28% NH_3_·H_2_O was added. The mixture was then diluted with 30 mL of deionized water and subjected to ultrasonic treatment for 10 min. Following this, 5 mL of manganese(II) acetylacetonate solution (25.3 mg, 0.1 mmol) was introduced, and the mixture was stirred and sonicated for another 10 min. The resulting mixture was transferred into a silica‐boron glass bottle and heated at 85 °C for 40 h. After cooling to room temperature, the black precipitate was separated by centrifugation and washed five times with DMF and acetone. The product was dried at 65 °C for 24 h, yielding MnPc‐Mn as a black powder with a 90% yield based on the initial amount of MnPc‐OH.

### Synthesis of Pt‐NPs/MnPc‐Mn

Initially, 40 mg of MnPc‐Mn was dispersed in 3 mL of deionized (DI) water. To synthesize Pt‐NPs/MnPc‐Mn, 10 mL of potassium tetrachloroplatinate (K_2_PtCl_4_, 2 mg mL^−1^) aqueous solution was added to the dispersion, followed by stirring for 1 h. Subsequently, 3 mL of Sodium borohydride (NaBH_4_) aqueous solution (1 mg mL^−1^) was added, and the mixture was stirred for 30 min to facilitate reduction. The resulting sample (10% Pt loading) was then centrifuged and washed three times with deionized water, and then dried under vacuum at 60 °C for 12 h. Pt loading of the hybrid catalyst was controlled by varying the amount of K_2_PtCl_4_ (e.g., 5 and 20 mL).

### Preparation of the Flexible Sensor Patch

For the diverse sensing functionalities, the flexible Au electrode was chosen as the working electrode (WE) substrate, which was fabricated on a polyimide (PI) base using cost‐effective and scalable photolithography and electroplating techniques.^[^ ^42^ ^]^ To fabricate the glucose sensor, the flexible Au electrode was modified with Pt‐NPs/MnPc‐Mn to directly catalyze glucose oxidation. The reference electrode (RE) was prepared by coating it with a commercial Ag/AgCl. To fabricate pH sensor, the aniline was electrochemically polymerized on Au surface to form a polyaniline (PANI) layer (Figure S17, Supporting Information). The RE was protected by a polyvinyl butyral (PVB) film saturated with NaCl to shield it from common interferences. To prepare the sealing membrane, 160 mg of PVB, 100 mg of NaCl, and 2 mg of F127 were dissolved in 2 mL of methanol. The resulting mixture was sonicated for 10 min, after which 4 µL of the solution was drop‐cast onto the Ag/AgCl‐coated reference electrode, followed by overnight curing in the dark. The temperature sensor was integrated into the sensor array using commercially available metal oxide components.^[^ ^43^ ^]^

## Conflict of Interest

The authors declare no conflict of interest.

## Supporting information



Supporting Information

## Data Availability

The data that support the findings of this study are available from the corresponding author upon reasonable request.
