# Supplementary material for: Sweat Wearable Sensor Based on Confined Pt Nanoparticles in 2D Conductive Metal–Organic Frameworks for Continuous Glucose Monitoring
Source: Adv Sci (Weinh). 2025 Jun 23;12(35):e07212. doi: 10.1002/advs.202507212 (PMC12463007; doi:10.1002/advs.202507212)
Supplement: Supplementary file 1 — Supporting Information [file ADVS-12-e07212-s001.docx]

Supporting Information

Sweat Wearable Sensor Based on Confined Pt Nanoparticles in 2D Conductive Metal-Organic Frameworks for Continuous Glucose Monitoring

*Wei Huang,^1^ Yong Yang,^3^ Yun Xu,^2^ Fei Xiao^1^*, Lin Wang^2^**

^1^Key Laboratory of Material Chemistry for Energy Conversion and Storage, Ministry of Education, School of Chemistry and Chemical Engineering, Huazhong University of Science & Technology, Wuhan 430074, China

^2^Union Hospital, Tongji Medical College, Huazhong University of Science & Technology, Wuhan 430022, China

^3^School of Mechanical Science and Engineering, Huazhong University of Science and Technology, Wuhan, 430074, China

E-mail: lin_wang@hust.edu.cn; xiaofei@hust.edu.cn

**Experimental Section**

*Materials and reagents:*

4,5-Dimethoxyphthalodinitrile was bought from Shanghai Haohong Biopharmaceutical Technology Co., Ltd. 2,3,6,7,10,11-hexahydroxytriphenylene, urea, Mn(acac)_2_, MnCl_2_, NaOH, aniline, and PVC were purchased from the Adamas-beta Co. Ltd. H_2_O_2_ (30%), (NH_4_)_6_Mo_7_O_24_·4H_2_O, KCl, NH_3_•H_2_O (25%~28%), K_2_HPO_4_·3H_2_O, KH_2_PO_4_ were purchased from the Macklin Co. Ltd. K_3_Fe(CN)_6_ and K_4_Fe(CN)_6_·3H_2_O were purchased from Sinopharm Chemical Reagent Co., Ltd. Ascorbic acid (AA), UA, DL-lactic acid (Lac), 3-hydroxytyramine hydrochloride (DA·HCl), glucose (Glu), glutathione reductase (GSH), ethanol (EtOH) and DMF, were bought from Aladdin Co. Ltd. CCK-8 Cell Counting Kit was purchased from Nanjing Vazyme Biotech Co., Ltd. Calcein/PI cell viability and cytotoxicity detection kit and penicillin/streptomycin double antibody (100×) were bought from Beyotime Biotechnology Co., Ltd. DMEM medium and fetal bovine serum were purchased from Gibco Life Technologies. Human immortalized keratinocyte cells (HACAT) were obtained from the Institute of Biochemistry and Cell Biology, Chinese Academy of Sciences. All reagents and solvents were used directly without further purification.

*Instruments:*

SEM image were obtained using a Nova NanoSEM 450 at 10 kV. TEM image were acquired using a Tecnai G2-F20 at 200 kV. HAADF-STEM image were acquired using FEI talos f200x, America. PXRD was carried out using a Rigaku D/max-IIIA diffractometer (Cu Kα, λ = 1.54056 Å). XPS measurements were tested using a Perkin-Elmer model PHI 5600 XPS system, with all peaks corrected by default for the C 1s line at 284.6 eV. The mechanical parameters were measured using a SHENZHEN SUNS Tester in the tensile mode, with a loading rate of 0.5 mm/min and a gauge length of 1.0 cm, equipped with a 1 N load cell. Specific surface areas were measured by the BET method, using a Quantachrome Instruments Autosorb-iQ2-MP at 77 K. Fourier transform infrared spectroscopy (FT-IR) measurements were performed by transmission through KBr pellets containing approximately 1% of the compounds using a PE Spectrum FT-IR spectrometer (400-4000 cm^-1^). Elemental analyses were performed by a Elementar VARIO Micro-Cube EL CHNS elemental analyzer. The Thermo Scientific HERACELL 150i was used as the cell culture incubator. The Thermo Scientific 1300 SERIES A2 was used as the biological safety cabinet. The optical microscope used was the Olympus CKX41 from Japan. The Olympus CX23 was used as the inverted fluorescence microscope. The PerkinElmer Victor NivoTM was used as the multifunctional microplate reader.

*Synthesis of* *(2,3,9,10,16,17,23,24-octamethoxyphthalocyaninato) manganese (II) (MnPc-OCH_3_).*

To a 100 mL round bottom flask was added 1 g (5.32 mmol) of 4,5-Dimethoxyphthalodinitrile and 200 mg of manganese chloride. 30 mL of anhydrous ethylene glycol was added with (NH_4_)_6_Mo_7_O_24_•4H_2_O (120 mg) and 60 mg of urea. The mixture was refluxed at 200 °C under nitrogen atmosphere for 3 days. Then, the reaction was quenched with approximately 50 mL of water and left stirring for 10 minutes. The precipitate was collected by centrifugation and washed successively with methanol and acetone for 3 times, respectively. The product was then dried in vacuum at 60 ºC. (yield 52%)

*Synthesis of 2,3,9,10,16,17,23,24-octa-hydroxylphthalocyanine manganese (II) (MnPc-OH).*

700 mg (1 mmol) of (2,3,9,10,16,17,23,24-octamethoxyphthalocyaninato) manganese (II) was suspended in 10 mL of anhydrous CH_2_Cl_2_. The reaction was stirred under nitrogen at room temperature for 30 minutes. 25 mL (25 eq.) of BBr_3_ (17% in CH_2_Cl_2_) was added slowly and the reaction was stirred for 1 week under N_2_. Then, 50 mL of methanol was slowly added into the mixture to quench unreacted BBr_3_. The mixture was stirred for 5 min and collected by centrifugation to remove the DCM. The precipitate was washed with DI water (3 × 20 mL) and acetone/ H_2_O mixture (3 × 15 mL until the supernatant became almost transparent. The final product was dried in a vacuum oven (60 ºC) and stored under nitrogen (yield 45%). ^1^H NMR (600 M, DMSO-d6): δ = 8.56 (s, 1H), 10.33 (br, 1H)..

*In vitro biocompatibility test*

*Cell culture:* The human immortalized keratinocyte cells (HACAT) were cultured in DMEM medium containing 10% fetal bovine serum and 1% penicillin/streptomycin at 37 °C in a humidified incubator with 5% CO_2_. All cell experiments were performed in a biological safety cabinet, and experimental materials and equipment were disinfected by UV irradiation for 30 min prior to use.

*Live/Dead Cell Staining:* HACAT cells were seeded at a density of 50,000 cells per well in 24-well plates and cultured for 24 h to allow complete attachment. The original medium was then removed from the wells and replaced with electrode-containing medium for 72 h in an incubator. The cells were then rinsed with PBS and incubated for 30 min at 37 °C with 2 μM calcein AM/PI staining solution prepared in fresh serum-free medium. Images were then captured using an inverted fluorescence microscope, processed and analysed using ImageJ.

*Cell Viability Assay Using CCK-8 Method:* HACAT cells were seeded in a 96-well plate at a density of 5,000 cells per well and cultured in an incubator for 24 h to allow complete adhesion. The medium was then replaced with electrode-containing medium (n = 5). PBS was used as a blank control, and fresh medium served as the negative control. After 24 h, the culture medium was removed, and the cells were rinsed with PBS. To prepare a 1:10 CCK-8 solution, add 100 μL of serum-free fresh medium to each well. Incubate the plate in a cell culture incubator for 1 h. After incubation, remove the 96-well plate and shake it on a shaker for 1 min. Then, place the plate in a microplate reader to measure the absorbance (A) at 450 nm for each well. Calculate cell viability based on the absorbance values (A) for each well using the formula: Cell viability (%) = (A_experiment_ - A_blank_) / (A_control_ - A_blank_) × 100%. Finally, generate a graph using GraphPad 8.

*On-body sweat analysis:*

All human trials were approved by the Medical Ethics Committee of Tongji Medical College, Huazhong University of Science and Technology, and written informed consent was obtained ([2024] Ethical Number: S104). A healthy subjects (males) aged 20-30 years were evaluated in vivo for glucose in sweat. After arriving at the laboratory, the subjects fasted overnight and then underwent sweat monitoring for 12 hours. During the human trials, data from the sensor patch was sent wirelessly via Bluetooth to the user interface. Meanwhile, sweat samples were periodically collected from the subject's forehead and neck using a centrifuge tube and then centrifuged at 6000 rpm for 15 min. The sweat samples were then frozen at -20 °C for further testing and validation by electrochemical testing of the wearable sensors, high-performance liquid chromatography analysis (Agilent), and commercial kits (Sinocare).


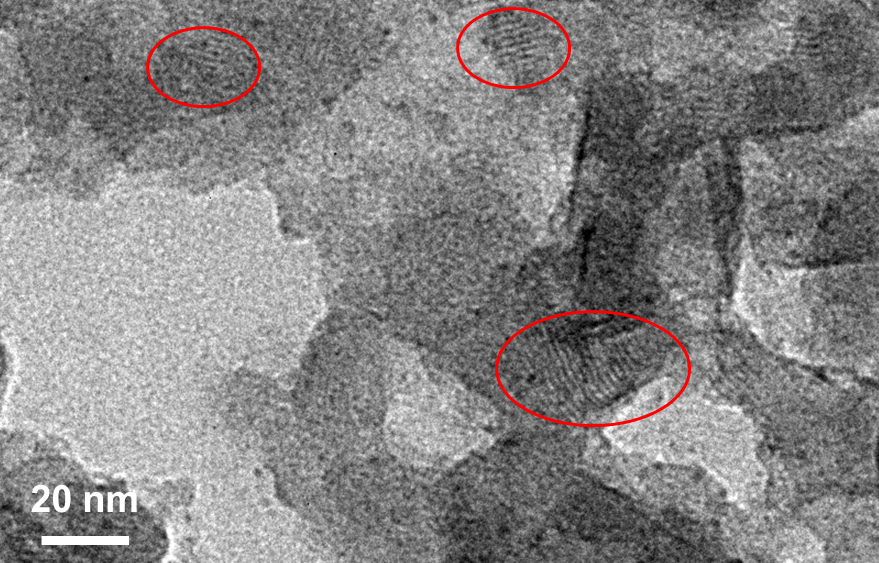


**Figure S1.** TEM spectrum of Pt-NPs/MnPc-Mn, from which we can see the obvious lattice fringes of MnPc-Mn.


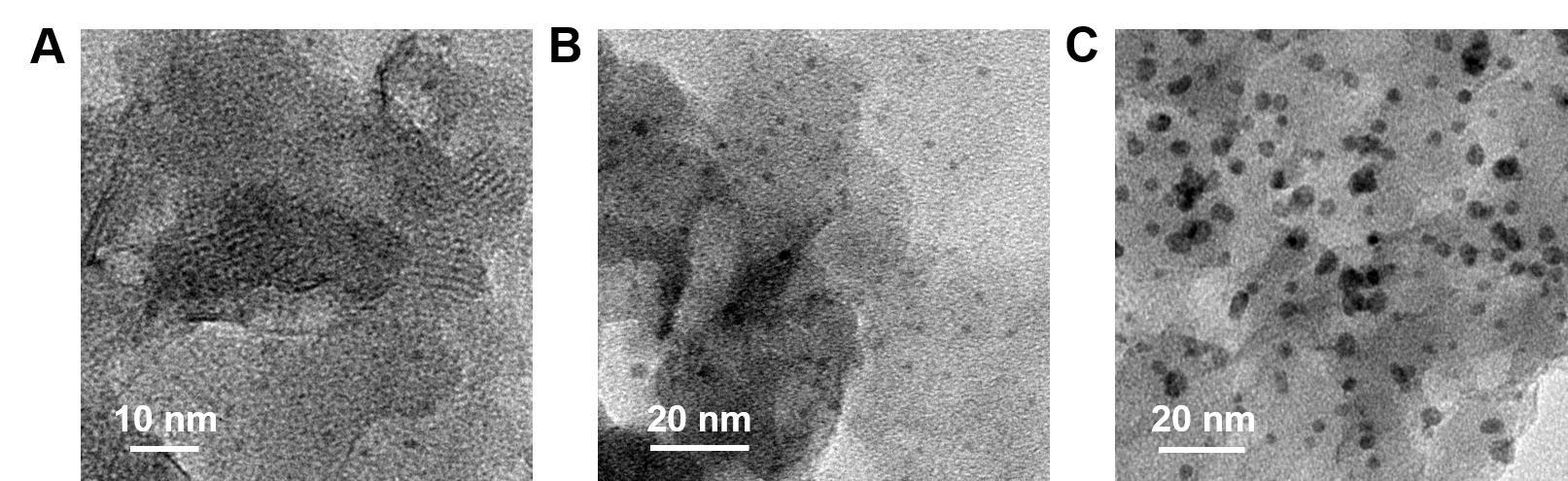


**Figure S2.** TEM images of Pt-NPs/MnPc-Mn synthesized with different platinum loadings: (A) 5 wt % Pt, (B) 10 wt % Pt, and (C) 20 wt % Pt.

**Figure S3.** Pore size distribution of Pt-NPs/MnPc-Mn and MnPc-Mn.


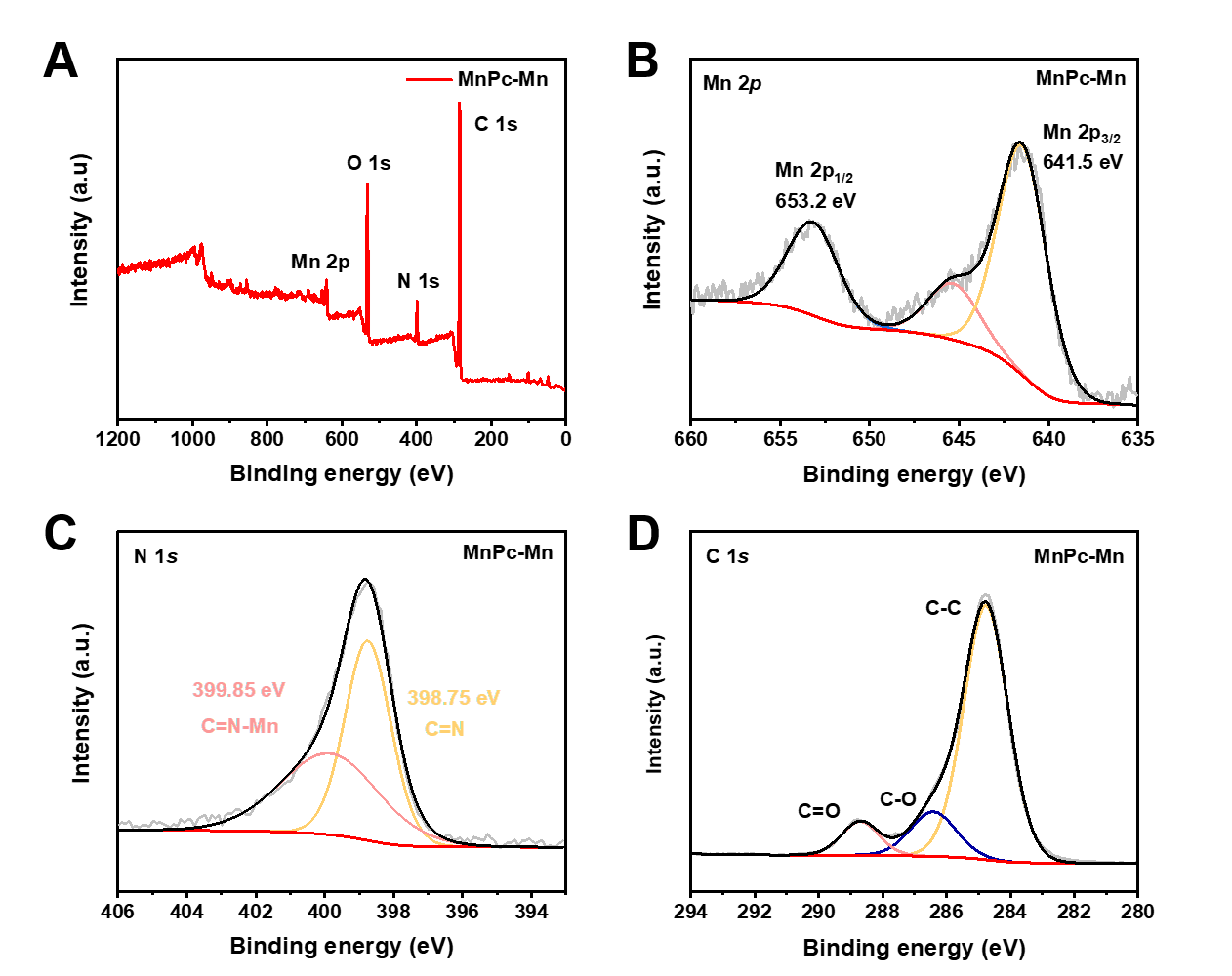


**Figure S4.** XPS analysis of pristine MnPc-Mn. (a) XPS survey spectrum of MnPc-Mn. (b) Mn 2p XPS spectrum of MnPc-Mn. (c) N 1s XPS spectrum of MnPc-Mn. (d) C 1s XPS spectrum of MnPc-Mn.

**
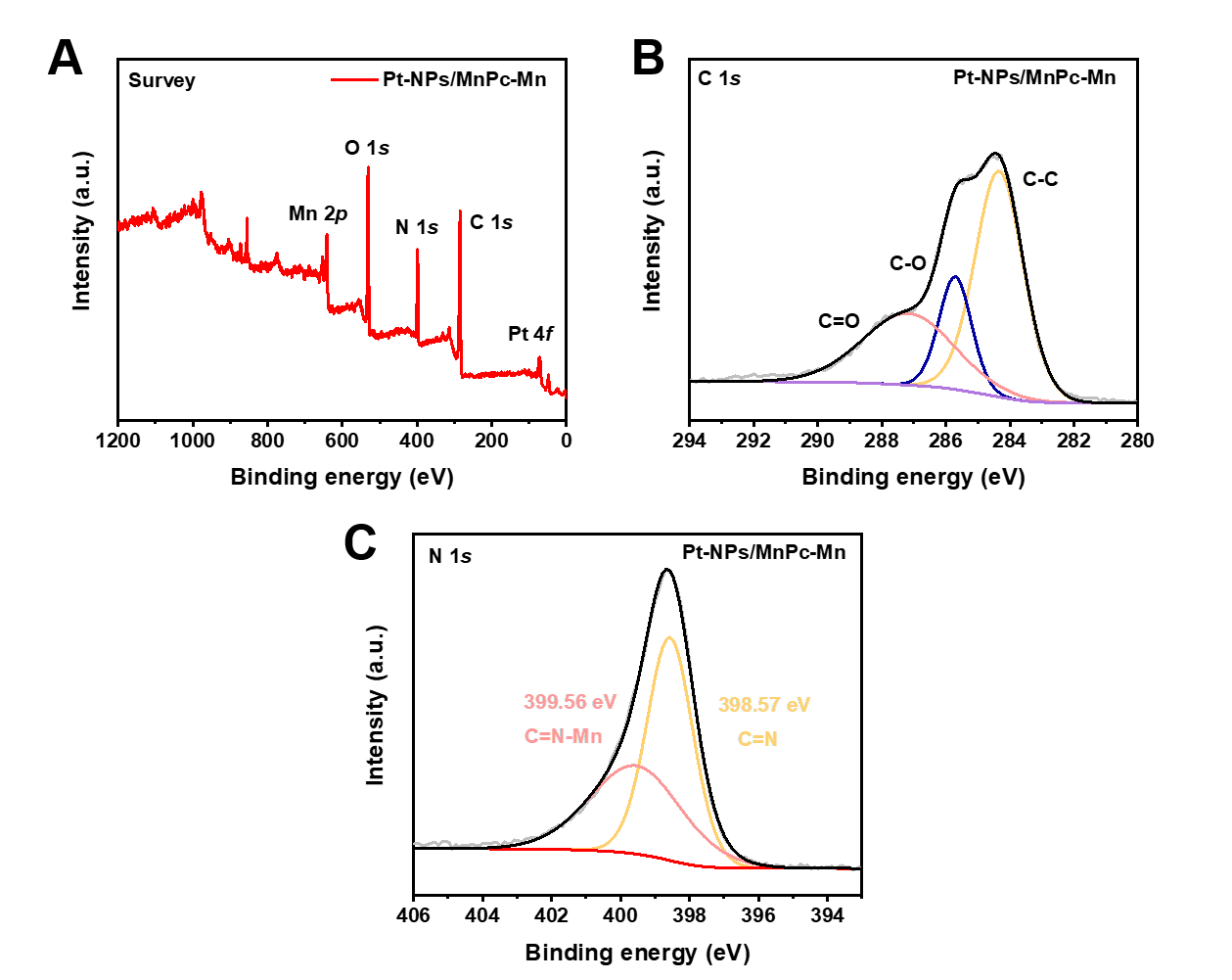
**

**Figure S5.** XPS analysis of Pt-NPs/MnPc-Mn. (a) XPS survey spectrum of Pt-NPs/MnPc-Mn. (b) C 1s XPS spectrum of Pt-NPs/MnPc-Mn. (c) N 1s XPS spectrum of Pt-NPs/MnPc-Mn.

**Figure S6.** Amperometric responses of Pt-NPs/MnPc-Mn/Au electrodes with varying Pt loadings upon injection of equal aliquots of glucose in 0.1 M PBS.


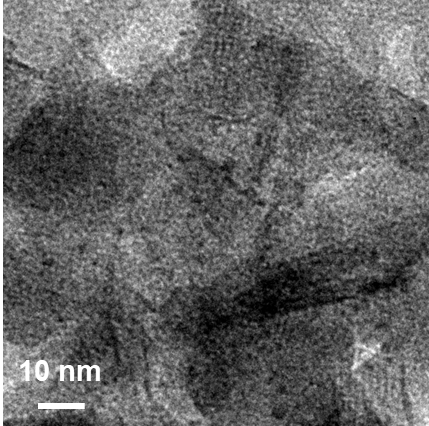


**Figure S7.** TEM spectrum of Pt-NPs/MnPc-Mn after 100 CV cycles in PBS (pH 7.0) solution containing glucose.

**Figure S8.** PXRD patterns of of Pt-NPs/MnPc-Mn before and after 100 continuous CV cycles in PBS.

**
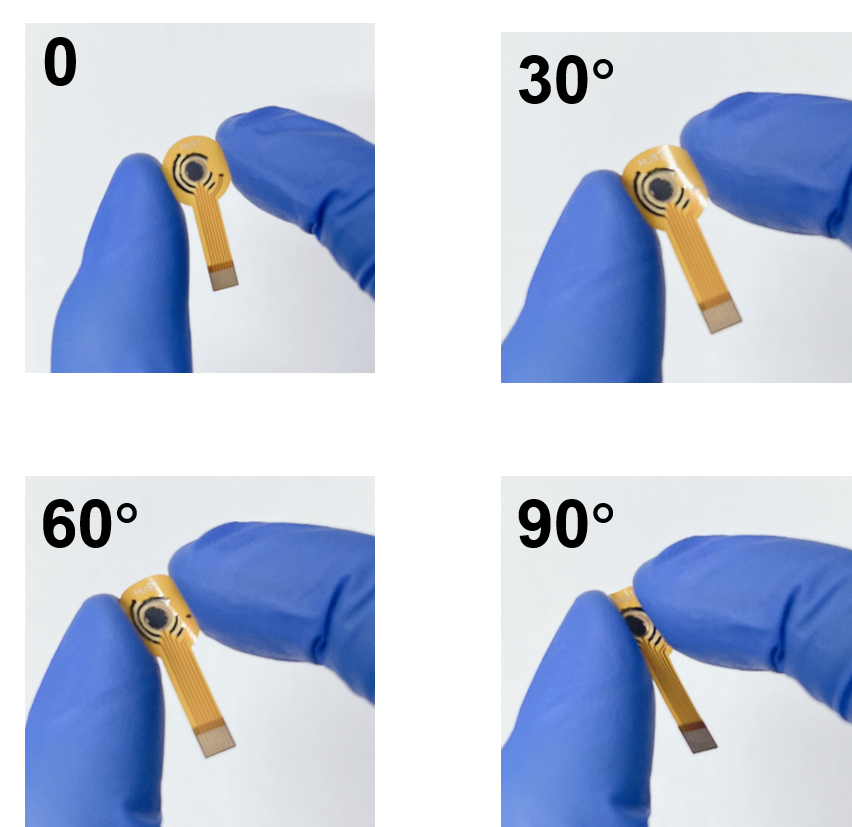
**

**Figure S9.** S Patch bending images with different angels.


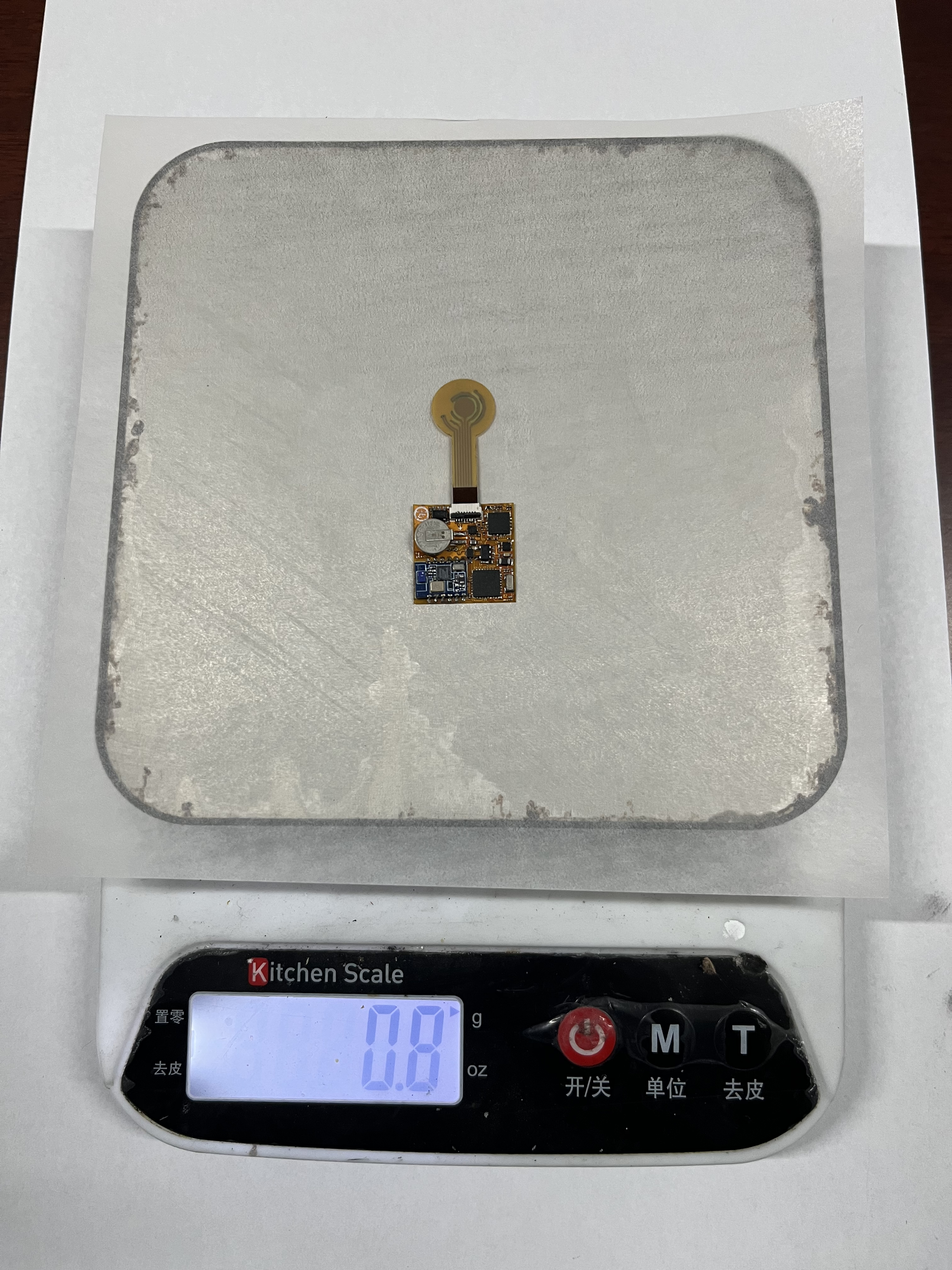


**Figure S10.** The weight of the fully integrated wearable sensor.

**
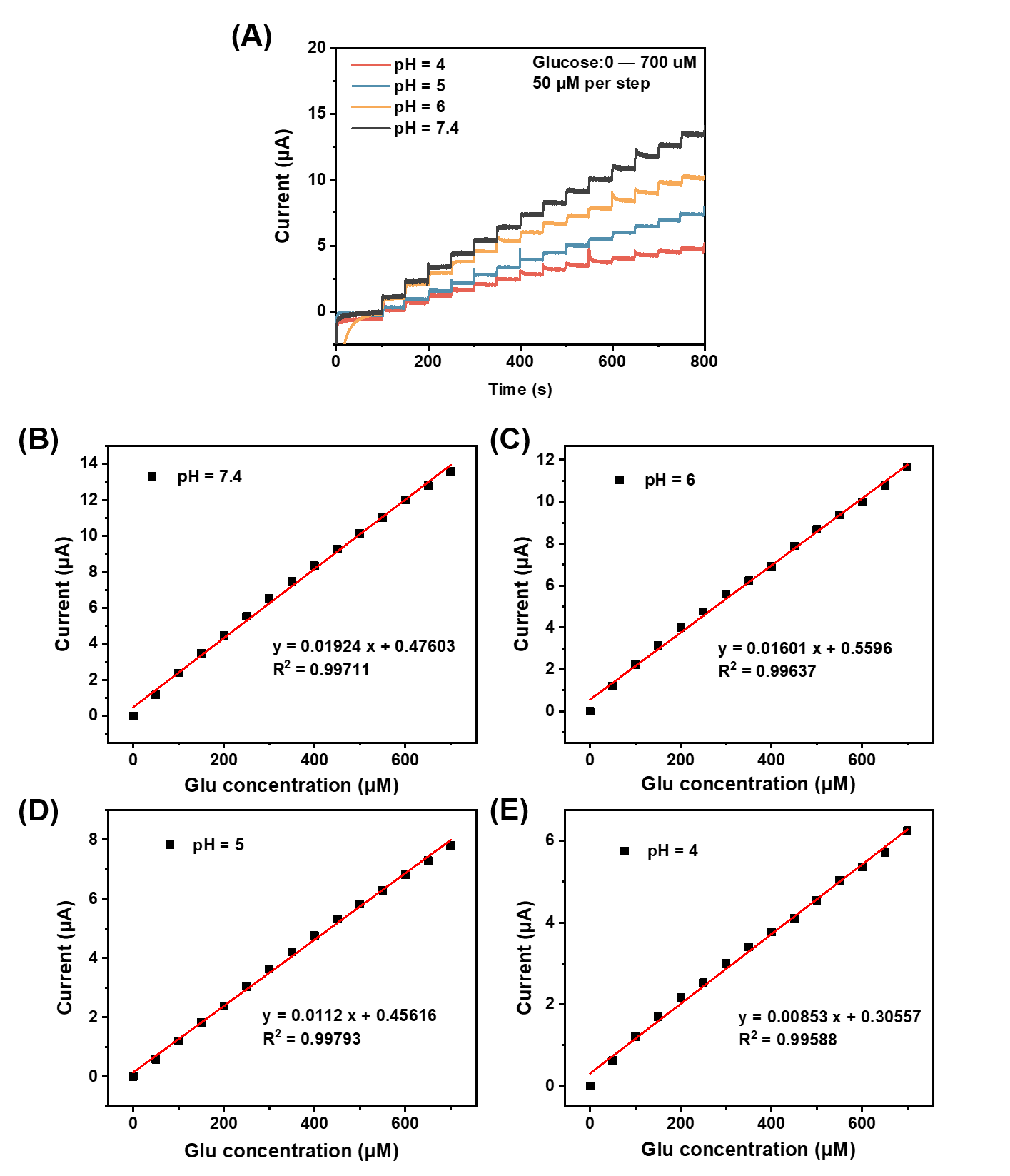
**

**Figure S11.** (A) Dynamic responses of the glucose sensor at decreasing pH in artificial sweat required for determining the pH correction factor. (B)-(E) Linearity curves of the glucose biosensor for different pH levels of artificial sweat.

**
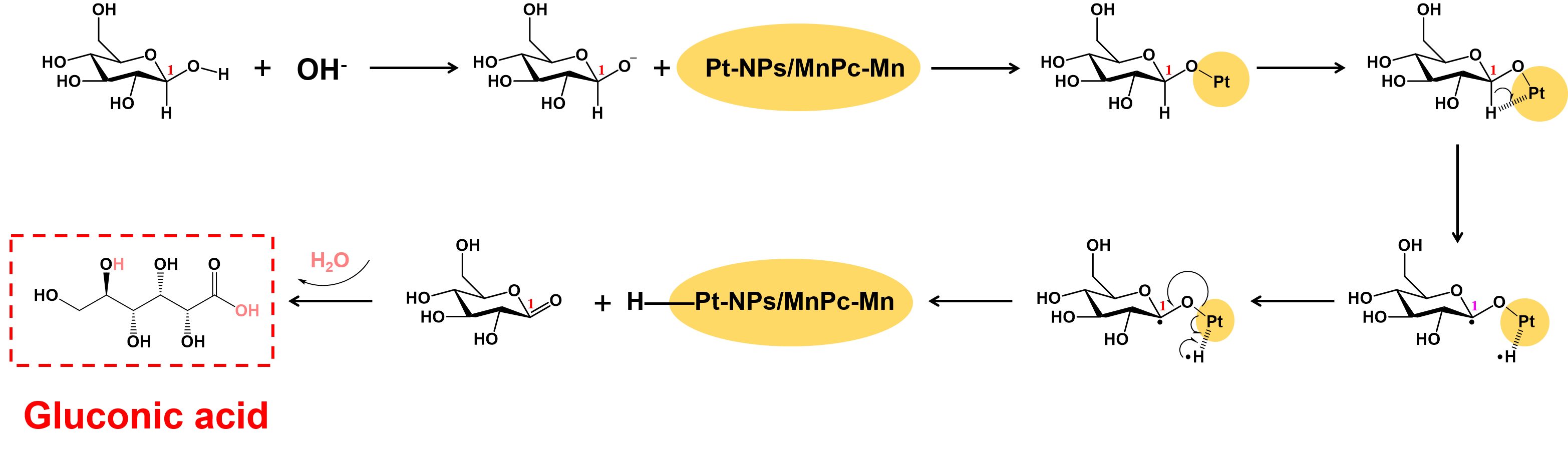
**

**Figure S12.** The charge transfer of Pt-NPs/MnPc-Mn catalyzed glucose oxidation.

**
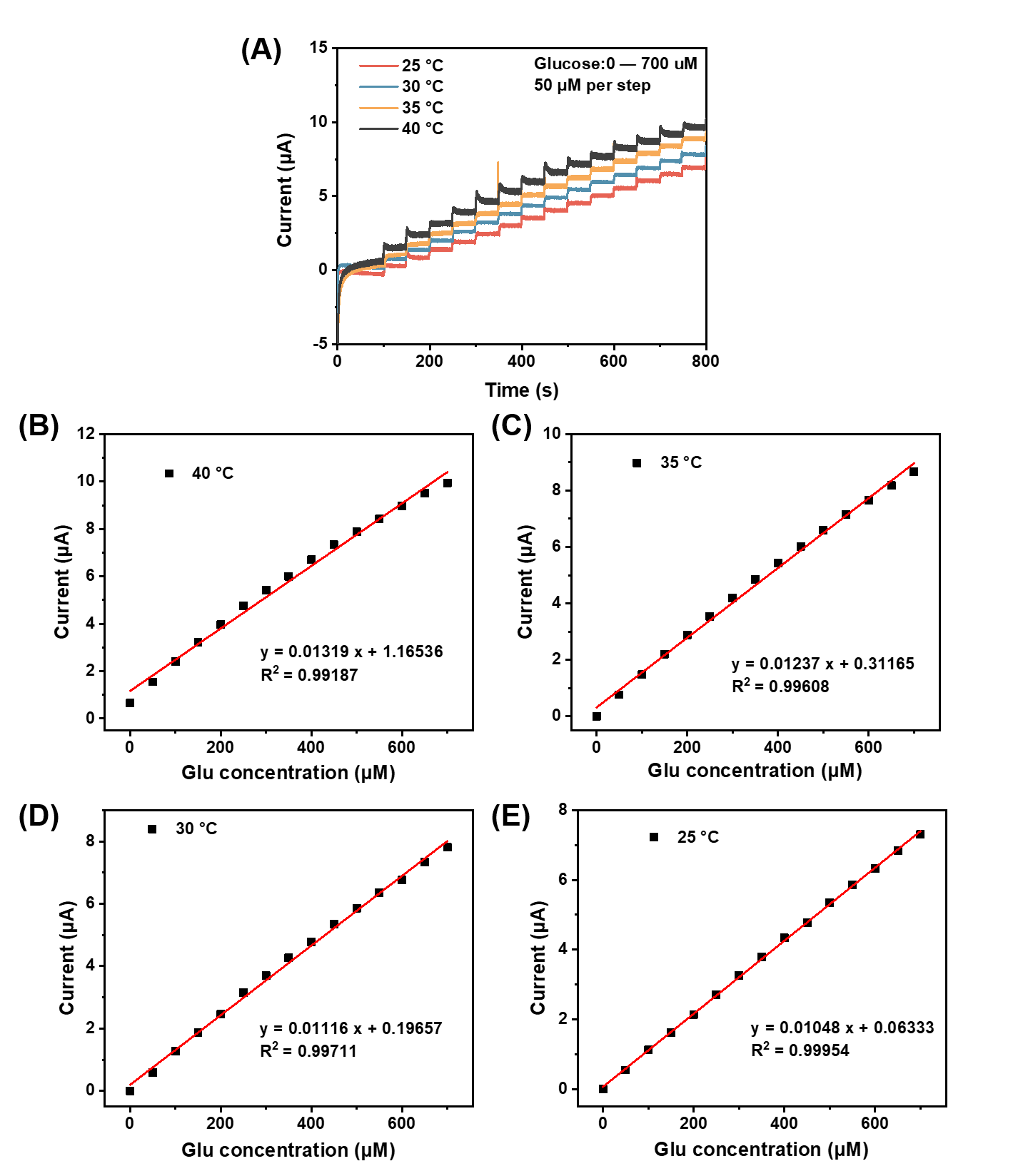
**

**Figure S13.** (A) Dynamic responses at different temperatures in artificial sweat (pH 6.5) required for determining the T correction factor. (B-E) Linearity curves of the glucose biosensor at increasing temperatures of artificial sweat.

**
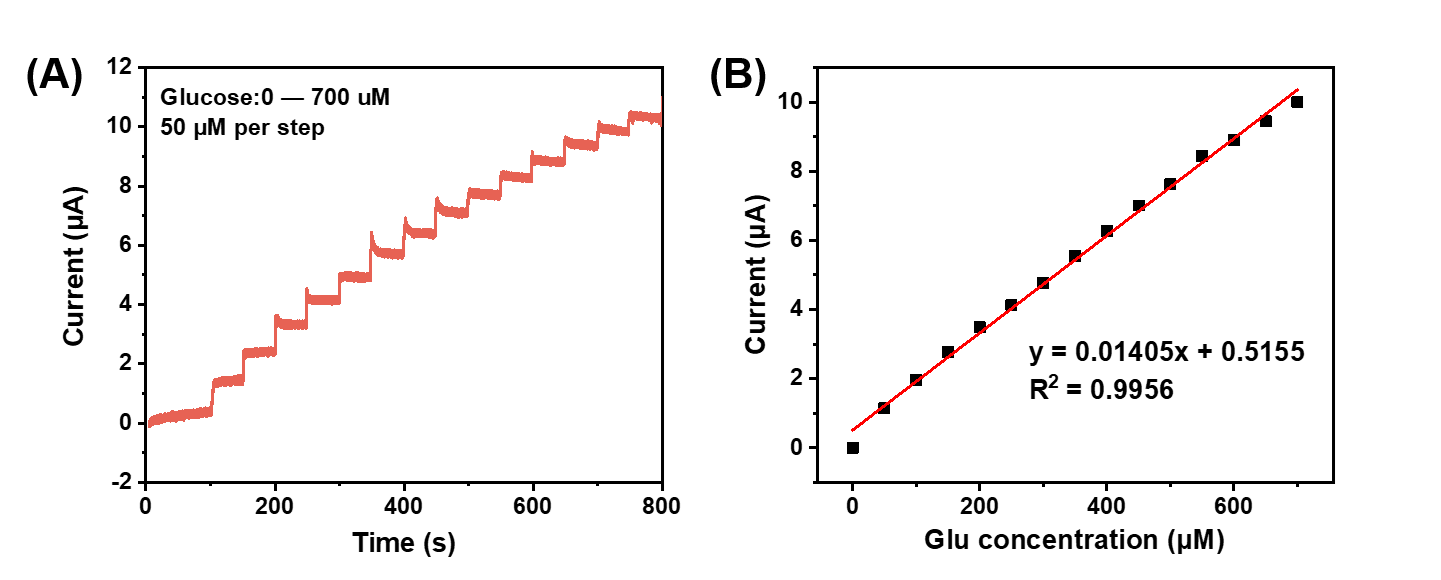
**

**Figure S14.** (A) Dynamic response in artificial sweat (pH 6.5, T = 20 °C) to determine the reference calibration equation for human perspiration. (B) Corresponding calibration graphs.


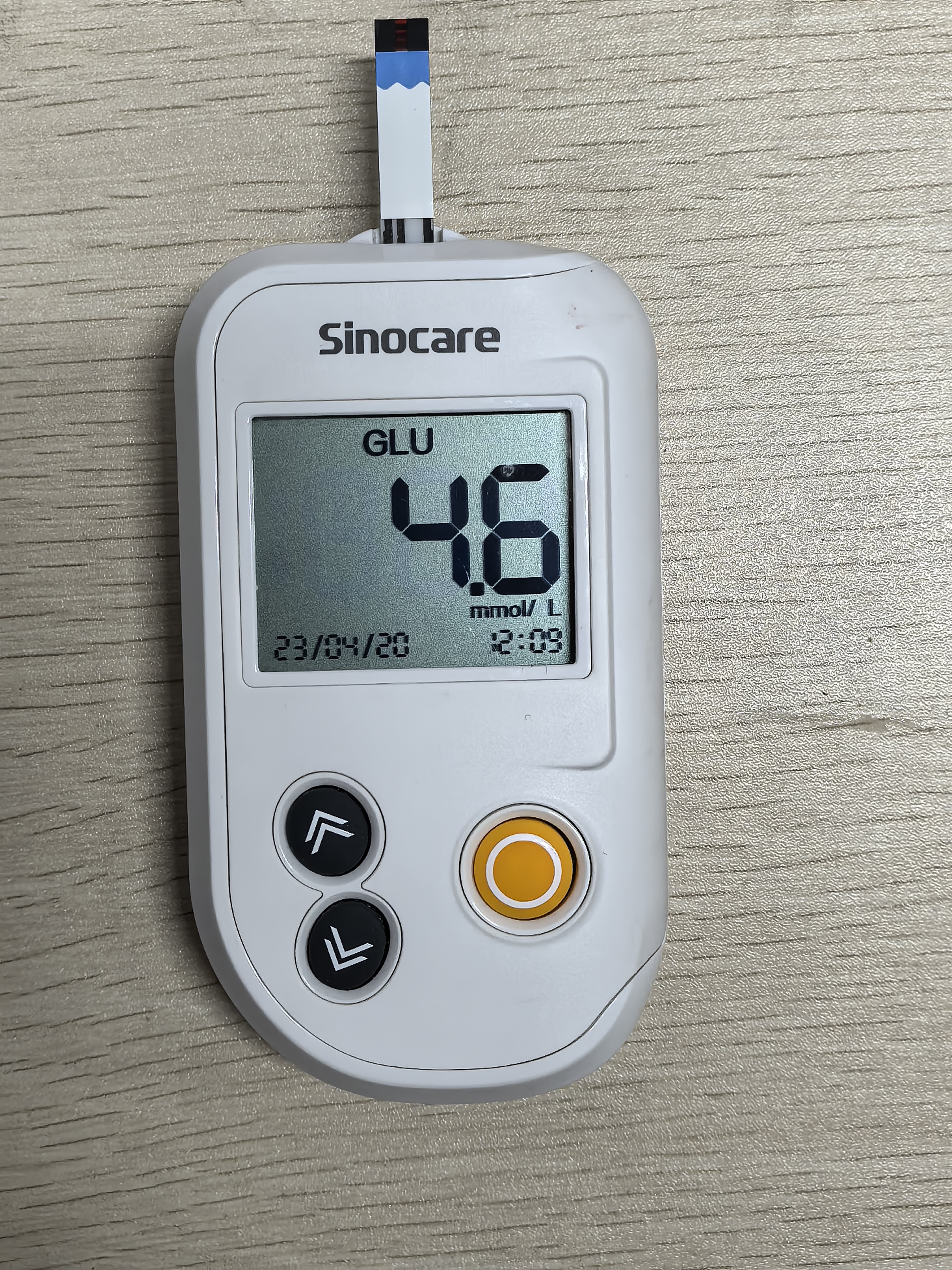


**Figure S15.** Testing blood glucose levels using a commercial glucose kit.

**Figure S16.** Correlation between blood glucose concentration and sweat glucose concentration (data lagged 20 minutes).

**Figure S17.** CV of 50 segments for electrodeposition of PANI on Au electrode.

**Table S1**. Comparison of the electrochemical sensing performance of Pt-NPs/MnPc-Mn versus recently reported nanocatalyst-based non-enzymatic glucose sensors under neutral conditions.

| Sample | Linear range | LOD  (μM) | Sensitivity  (μA mM^-1^ cm^-2^) | Ref. |
| --- | --- | --- | --- | --- |
| Pt-NPs/MnPc-Mn | 5~ 800 μM | 5.5 | 25.48 | This work |
| Au/CNTs | 0.1~25 mM | 14.1 | 2.77 | 1 |
| Pd@Pt | 1~8.5 mM | 0.82 | 15.14 | 2 |
| Pt nano pine needles | 0.1~4 mM  and  5~10 mM | 14.77 | 20.31 | 3 |
| CoFe PB | 0.1~8.2 mM | 67 | 18.69 | 4 |
| AuNPs modified  laser-induced  graphene | 10 µM ~10 mM | 26 | 0.024 | 5 |
| CoWO_4_/CNT coated onto the CNT-AuNS | 0.05~0.3 mM | 13 | 10.89 | 6 |
| Fluorocarbon-based  materials coated on  Au | 30 µM ~1.1 mM | 15 | 11.4 | 7 |
| AuNs-LSGE | 0.5~20 mM | 210 | - | 8 |
| PMH | 0.58~3.08 mM | 82 | - | 9 |
| Ni/Pt-BDD | 2-12 mM | 87.1 | 18.5 | 10 |
| Rhodium oxide doped Au | Up to 3 mM | 20 | 3.52 | 11 |
| Glucose oxidase/bilirubin oxidase (BOD)/highly porous gold(hPG) | 50 μM~1mM | 50 | 14.13 | 12 |

**Table S2**. Comparative long‑term stability of the Pt‑NPs/MnPc‑Mn‑based sensor versus other electrochemical glucose sensors.

| Sample | Long-term stability | Ref. |
| --- | --- | --- |
| Pt-NPs/MnPc-Mn | 100 days (93%) | This work |
| PtNi alloy | 4 weeks (90%) | 13 |
| PdAg NPs | 60 days (93%) | 14 |
| PdAg NWs | 30 days | 15 |
| Au@Pt/Au NPs | 27 days (94.3%) | 16 |
| Au/NiAu NWs | 20 days (92%) | 17 |
| PtPd/rGO | 1 month (92.95%) | 18 |
| rGO/PU/Au | 7 days (stable) | 19 |
| Pt/MCs | 20 days (stable) | 20 |
| AuNPs/PANI/CC | 15 days (95%) | 21 |
| PtPd/nanopores carbon | 2 weeks (92.6%) | 22 |
| PtAu-MnO_2_ | 30 days (95%) | 23 |
| Cu_3_Pt/Cu_2_O | 10 days (95.33%) | 24 |

**Table S3**. Relationship between the volume of sweat in the pipette tip and the sweat remaining in the pipette.

| Sweat volume in the pipette tip (μL) | Whether there is biofluid obviously left in the pipette tip after sampling ^a^ |
| --- | --- |
| 2.4 | No |
| 2.5 | No |
| 2.6 | Yes |
| 2.4 | No |
| 2.5 | No |
| 2.6 | Yes |
| 2.4 | No |
| 2.5 | No |
| 2.6 | Yes |

^a^ Observed by naked eyes.

**Table S4.** The cost analysis of the wearable sensor device.

| **Material** | **Single cost** | **Amount/Sensor** | **Cost/Sensor ($)** |
| --- | --- | --- | --- |
| MnPc-Mn@Pt-NPs | $150 g^-1^ | 0.5 mg | 0.075 |
| NBG | $25 g^-1^ | 1 mg | 0.025 |
| Ag/AgCl | $200 g^-1^ | 0.1 mg | 0.02 |
| PDMS | $0.175 g^-1^ | 2 g | 0.35 |
| aniline | $0.02/mL | 0.1 mL | 0.002 |
| NiMnO_3_ | $0.5 | - | 0.5 |
| **Sensor patch total** | - | - | **~0.97** |
| **Reuseable microfluidic system** | - | - | **3** |
| **Reuseable readout PCB** | - | - | **~20** |

**Table S5.** The cost of the wearable sensor device and Commercial Blood Glucose Meter.

| **brand** | **Cost ($)** |
| --- | --- |
| This work | 24 |
| Abbot | 46 |
| Sinocare | 52 |
| Yuwell | 40 |
| Silicon | 43 |
| Microtech Medical | 42 |

1. **Glucose Biosensor Calibration for pH and Temperature Variation**

The responses and calibration equations in artificial sweat (pH = 6.5, temperature = 20 °C) are shown in Figure S10, which are used to establish reference calibration equations for human sweat. Since the effects of pH and temperature on the glucose biosensor are cumulative, the slope (M) and intercept (K) of the calibration equation in artificial sweat at pH 6.3 and 20 °C were used to recalculate glucose concentration for reference. The corresponding glucose concentration was calculated using the linear equation from Figure S10B. Therefore, by following a correction algorithm, we computed the corrected slope (Kcorrected) and intercept (Mcorrected), which were ultimately used to determine the precise glucose concentration in sweat during in vivo tests. The calibration method for accurate glucose measurement based on pH and temperature variations is described in detail below.

Glucose biosensor linearity equation in artificial sweat (pH = 6.5, T = 20 °C) from Figure 10B

y = 0.01405x +0.5155···································································(S1)

where y = current in μA, and x = glucose concentration in μM.

pH sensor linearity equation in 0.1 M PBS from Figure 4B

OCP = -40.792 pH + 383.74····························································(S2)

where OCP = open circuit potential response in mV, and pH = value of pH of the PBS solution

Temperature sensor linearity equation from Figure 4G

V = 0.03 T + 0.822········································································(S3)

where V = Voltage of the temperature sensor in V and T = temperature in °C.

Here, we explain the calculation method for correcting glucose concentration based on variations in pH and temperature. It is assumed that the effects of pH and temperature on glucose oxidase are independent of each other. Therefore, the variation coefficients for each condition relative to the reference conditions of pH 6.5 and temperature 20 °C (including the pH-dependent P_slope_ and P_intercept_, and the temperature-dependent T_slope_ and T_intercept_) can be calculated. This allows for the independent assessment of how pH and temperature affect the slope and intercept of each glucose biosensor. The following formulas are used to express these effects:

*Pslope* = ········································································(S4)


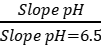


*Pintercept* = ································································(S5)


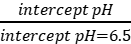


*Tslope* = ···········································································(S6)


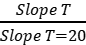


*Tintercept* = ·······································································(S7)


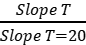


**Table S6** The correction factors for the slope and intercept calculated at different pH values from the data collected in Figure S7 and using Equations S4 and S5.

| pH | Slope | P_slope_ | Intercept | P_intercept_ |
| --- | --- | --- | --- | --- |
| 4 | 0.00853 | 0.60711744 | 0.30557 | 0.59276431 |
| 5 | 0.0112 | 0.79715302 | 0.45616 | 0.88488846 |
| 6 | 0.01601 | 1.13950178 | 0.5596 | 1.08554801 |

Linear equations of pH corresponding to P_slope_ and P_intercept_

P*_slope (c)_* = 0.26619 pH -048304, R^2^ = 0.97344·········································(S8)

P*_intercept (c)_* = 0.24639 pH -0.37756, R^2^ = 0.98865 ·····································(S9)

**Table S7**. The correction factors for the slope and intercept calculated at different temperatures from the data collected in Figure S9 and using Equations S6 and S7.

| Temperature, T(°C) | Slope | T_slope_ | Intercept | T_intercept_ |
| --- | --- | --- | --- | --- |
| 30 | 0.01116 | 0.79431 | 0.19657 | 0.38132 |
|  |  |  |  |  |
| 35 | 0.01237 | 0.88043 | 0.31165 | 0.60456 |
| 40 | 0.01319 | 0.93879 | 1.16536 | 2.26064 |

Linear equations of temperature corresponding to Tslope and Tintercept

T*_slope (c)_* = 0.01445 T + 0.36548, R^2^ = 0.98785 ·········································(S10)

T*_intercept (c)_* = 0.18793 T – 5.49545, R^2^ = 0.83769······································ (S11)

Using Equations S8, S9, S10, and S11, we can determine the slope factors (P*_slope(c)_* and T*_slope (c))_* and intercept factors (P*_intercept (c)_* and T*_intercept (c))_*.

Considering the cumulative effects of pH and temperature, the entire response of the biosensor can be corrected by recalculating the slope and intercept (denoted as K and M, respectively) of the initial in vitro calibration curve in artificial sweat, using pH 6.5 and 20 °C as reference conditions. Therefore, we calculated the final corrected slope (K_corrected_) and intercept (M_corrected_), which are used to accurately determine the glucose concentration in sweat.

*K_corrected_* = *K* × *P_slope (c)_* × *T_slope (c)_* ·························································(S12)

*M_corrected_* = *M* × *P_intercept (c)_* × *T_intercept (c)_* ··················································(S13)

Next, the corrected slope and intercept are calculated using Equations (S12) and (S13), taking into account the initial linear calibration curve of the glucose biosensor (with pH 6.5 and 20 °C, where the slope is K and the intercept is M).

The final step is to calculate the blood glucose concentration by taking into account the corrected slope (*K*_corrected_) and intercept (*M*_corrected_) of the calibration plot and the current value measured at the body surface.

𝐶𝑜𝑛𝑐𝑒𝑛𝑡𝑟𝑎𝑡𝑖𝑜𝑛 𝑜𝑓 𝑔𝑙𝑢𝑐𝑜𝑠𝑒 = μM······················(S14)


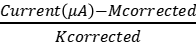


**Reference**

[1] D. Branagan, C. B. Breslin, *Sens. Actuators, B* **2019**, *282*, 490.

[2] Y. S. Wu, Z. W. Wu, C. L. Lee, *Sens. Actuators, B* **2019**, *281*, 1.

[3] G. Luo, J. Liu, J. Xie, W. Jing, M. Li, L. Zhao, Z. Li, P. Yang, Z. Jiang, *Dalton Trans*. **2023**, *52*, 12988.

[4] R. C. Oglou, T. G. U. Ghobadi, E. Ozbay, F. Karadas, *Anal. Chim. Acta* **2021**, *1188*, 339188

[5] C. W. Bae, P. T. Toi, B. Y. Kim, W. I. Lee, H. B. Lee, A. Hanif, E. H. Lee, N. E. Lee, *ACS Appl. Mater. Interfaces*, **2019**, *11*, 14567.

[6] S. Y. Oh, S. Y. Hong, Y. R. Jeong, J.Yun, H. Park, S. W. Jin, G. Lee, J. H. Oh, H. Lee, S. S. Lee, J. S. Ha, *ACS Appl. Mater. Interfaces*, **2018**, *10*, 13729.

[7] X. Zhu, Y. Ju, J. Chen, D. Liu, H. Liu, *ACS Sens*, **2018**, *3*, 1135.

[8] A. Berni, A. Amine, J. J. García-Guzmán, L. Cubillana-Aguilera, J. M. Palacios-Santander, *Biosensors* **2023**, *13*, 678.

[9] D. Lu, H. Li, N. Xiao, M. Jiang, Y. Zuna, S. Feng, Z. Li, J. Long, J. L. Marty, Z. Zhu, *Talanta* **2025**, *283*, 127197.

[10] R. Zhu, Z. Zhao, J. Cao, H. Li, L. Ma, K. Zhou, Z. Yu, Q. Wei, *J. Electroanal. Chem.* **2022**, *907*, 116084.

[11] W. Xiao, M. Li, D. Li, B. Shi, R. Zhong, Y. Zhao, Q. Tai, S. He, Q. Dong, *Langmuir* **2024**, *40*, 20797.

[12] C. Gonzalez-Solino, E. Bernalte, C. B. Royo, R. Bennett, D. Leech, M. D. Lorenzo, *ACS Appl. Mater. Interfaces* **2021**, *13*, 26704.

[13] R. Wang, X. Liang, H. Liu, L. Cui, X. Zhang, C. Liu, *Mikrochim. Acta* **2018**, 185, 339.

[14] S. Liu, C. Zhang, L. Yuan, J. Bao, W. Tu, M. Han, Z. Dai, *Part. Part. Syst. Charact.* **2013**, 30, 549.

[15] X. Xu, H. Lv, L. Sun, P. Song, B. Liu, X. Chen, *ChemPlusChem* **2020**, 85, 970.

[16] K. Shim, W. C. Lee, M.-S. Park, M. Shahabuddin, Y. Yamauchi, M. S. A. Hossain, Y. B. Shim, J. H. Kim, *Sens. Actuators, B.* **2019**, 278, 88.

[17] L. Qin, L. He, J. Zhao, B. Zhao, Y. Yin, Y. Yang, *Sens. Actuators, B* **2017**, 240, 779.

[18] M. Li, X. Bo, Y. Zhang, C. Han, L. Guo, *Biosens. Bioelectron.* **2014**, 56, 223.

[19] P. T. Toi, T. Q. Trung, T. M. L. Dang, C. W. Bae, N. E. Lee, *ACS Appl. Mater. Interfaces* **2019**, 11, 10707.

[20] C. Su, C. Zhang, G. Lu, C. Ma, *Electroanalysis* **2010**, 22, 1901.

[21] M. Xu, Y. Song, Y. Ye, C. Gong, Y. Shen, L. Wang, L. Wang, *Sens. Actuators, B* **2017**, 252, 1187.

[22] X. Bo, J. Bai, L. Yang, L. Guo, *Sens. Actuators, B* **2011**, 157, 662.

[23] F. Xiao, Y. Li, H. Gao, S. Ge, H. Duan, *Biosens. Bioelectron.* **2013**, 41, 417.

[24] B. Yang, N. Han, L. Zhang, S. Yi, Z. Zhang, Y. Wang, Y. Zhou, D. Chen, Y. Gao, *Appl. Surf. Sci.* **2020**, 534, 147596.
